# Characterization of a neutralizing antibody that recognizes a loop region adjacent to the receptor-binding interface of the SARS-CoV-2 spike receptor-binding domain

**DOI:** 10.1128/spectrum.03655-23

**Published:** 2024-02-28

**Authors:** Itsuki Anzai, Junso Fujita, Chikako Ono, Yoichiro Kosaka, Yuki Miyamoto, Shintaro Shichinohe, Kosuke Takada, Shiho Torii, Shuhei Taguwa, Koichiro Suzuki, Fumiaki Makino, Tadahiro Kajita, Tsuyoshi Inoue, Keiichi Namba, Tokiko Watanabe, Yoshiharu Matsuura

**Affiliations:** 1 Department of Molecular Virology, Research Institute for Microbial Diseases, Osaka University, Suita, Osaka, Japan; 2 Center for Infectious Disease Education and Research (CiDER), Osaka University, Suita, Osaka, Japan; 3 Graduate School of Frontier Biosciences, Osaka University, Suita, Osaka, Japan; 4 JEOL YOKOGUSHI Research Alliance Laboratories, Osaka University, Suita, Osaka, Japan; 5 Graduate School of Pharmaceutical Sciences, Osaka University, Suita, Osaka, Japan; 6 Laboratory of Virus Control, Research Institute for Microbial Diseases, Osaka University, Suita, Osaka, Japan; 7 Bio Matrix Research Inc., Nagareyama, Chiba, Japan; 8 Center for Advanced Modalities and DDS, Osaka University, Suita, Osaka, Japan; 9 The Research Foundation for Microbial Diseases of Osaka University (BIKEN), Suita, Osaka, Japan; 10 JEOL Ltd., Akishima, Tokyo, Japan; 11 RIKEN Center for Biosystems Dynamics Research and Spring-8 Center, Suita, Osaka, Japan; University of Arizona, Tucson, Arizona, USA

**Keywords:** severe acute respiratory syndrome-coronavirus 2 (SARS-CoV-2), monoclonal antibody, spike protein, receptor-binding domain, conformational transition, neutralizing epitope

## Abstract

Although the global crisis caused by the coronavirus disease 2019 (COVID-19) pandemic is over, the global epidemic of the disease continues. Severe acute respiratory syndrome coronavirus-2 (SARS-CoV-2), the cause of COVID-19, initiates infection via the binding of the receptor-binding domain (RBD) of its spike protein to the human angiotensin-converting enzyme II (ACE2) receptor, and this interaction has been the primary target for the development of COVID-19 therapeutics. Here, we identified neutralizing antibodies against SARS-CoV-2 by screening mouse monoclonal antibodies and characterized an antibody, CSW1-1805, that targets a narrow region at the RBD ridge of the spike protein. CSW1-1805 neutralized several variants *in vitro* and completely protected mice from SARS-CoV-2 infection. Cryo-EM and biochemical analyses revealed that this antibody recognizes the loop region adjacent to the ACE2-binding interface with the RBD in both a receptor-inaccessible “down” state and a receptor-accessible “up” state and could stabilize the RBD conformation in the up-state. CSW1-1805 also showed different binding orientations and complementarity determining region properties compared to other RBD ridge-targeting antibodies with similar binding epitopes. It is important to continuously characterize neutralizing antibodies to address new variants that continue to emerge. Our characterization of this antibody that recognizes the RBD ridge of the spike protein will aid in the development of future neutralizing antibodies.

**IMPORTANCE:**

SARS-CoV-2 cell entry is initiated by the interaction of the viral spike protein with the host cell receptor. Therefore, mechanistic findings regarding receptor recognition by the spike protein help uncover the molecular mechanism of SARS-CoV-2 infection and guide neutralizing antibody development. Here, we characterized a SARS-CoV-2 neutralizing antibody that recognizes an epitope, a loop region adjacent to the receptor-binding interface, that may be involved in the conformational transition of the receptor-binding domain (RBD) of the spike protein from a receptor-inaccessible “down” state into a receptor-accessible “up” state, and also stabilizes the RBD in the up-state. Our mechanistic findings provide new insights into SARS-CoV-2 receptor recognition and guidance for neutralizing antibody development.

## INTRODUCTION

Severe acute respiratory syndrome coronavirus 2 (SARS-CoV-2) emerged in Wuhan, China, in late 2019 (1–3) and rapidly spread around the world, causing a pandemic of coronavirus disease 2019 (COVID-19) that has resulted in more than 769 million confirmed cases and 6.9 million deaths, as of 9 August 2023 (4). SARS-CoV-2 infection is initiated by the binding of the spike protein on the virion surface to the host cell receptor, angiotensin-converting enzyme II (ACE2) (5–8). The spike protein comprises an S1 domain, consisting of an N-terminal domain (NTD) and a receptor-binding domain (RBD), and an S2 domain that contains a fusion peptide, with a furin cleavage site between S1 and S2 (6, 8). Structural analysis has revealed that the RBD has two conformations: “down” and “up” (6, 9). The conformational change of RBD from down to up exposes the interaction site of the RBD with ACE2, allowing the spike to bind to ACE2 (10–13). Then, the fusion peptide, exposed by protease cleavage at the furin cleavage site, is inserted into the host cell membrane, resulting in the fusion of the virus envelope with the cell membrane and the initiation of cell invasion (8).

Since the binding of the RBD with ACE2 is essential for SARS-CoV-2 infection, antibodies targeting the RBD are expected to be an effective treatment for COVID-19. Most of the neutralizing antibodies against SARS-CoV-2, including the approved antibody drugs (14–19), target the RBD (20, 21). Many structural and biophysical analyses have been performed on neutralizing antibodies against SARS-CoV-2 (22–24), leading to a wealth of knowledge on the subject. However, continuous detailed characterizations of neutralizing antibodies may be needed to respond to the emergence of new variants.

In this study, we generated mouse monoclonal antibodies against the SARS-CoV-2 spike and identified a neutralizing antibody, CSW1-1805, that recognizes the loop region adjacent to the ACE2 interaction interface with the RBD, the so-called RBD ridge. CSW1-1805 exhibited *in vitro* neutralizing activity against several variants, including Alpha, Beta, Gamma, and Delta, and completely protected mice from mouse-adopted SARS-CoV-2 infection. Cryo-EM and biochemical analysis showed that CSW1-1805 has a narrow binding epitope and that the binding of CSW1-1805 locked the RBD in the up conformation. Furthermore, a comparison of CSW1-1805 with previously reported antibodies that bind to the RBD ridge suggests that CSW1-1805 has different binding properties than those of previously reported antibodies, including complementarity determining regions (CDRs) with different characteristics. This report contributes to our knowledge of neutralizing antibodies that bind to the RBD ridge of the SARS-CoV-2 spike protein.

## RESULTS

### Screening of mouse monoclonal antibodies that neutralize VSV-pseudotyped SARS-CoV-2

To produce monoclonal antibodies that recognize the SARS-CoV-2 spike protein, we first prepared an antigen for mouse immunization: the ectodomain of the spike from the Wuhan strain (Spike^Wuhan^) with the furin cleavage site mutation and the tandem proline mutation (9) followed by the C-terminal foldon motif (25) and a His-tag, which was produced by a mammalian cell expression system (Fig. S1). Hybridomas were generated from mice immunized with purified Spike^Wuhan^ and then screened for clones producing anti-Spike^Wuhan^ antibodies, resulting in the identification of 70 clones (Fig. S2A and B). We next examined whether these antibody clones bound to the S1 domain, NTD, and/or RBD and found that 12 clones and 37 clones recognized the NTD and RBD, respectively (Fig. S2C). The remaining 21 clones did not bind to the S1 domain, NTD, or RBD, suggesting that they may recognize the S2 domain (Fig. S2C).

Next, we examined the inhibitory effect of the 70 identified clones on Spike^Wuhan^ and ACE2 binding by using an enzyme-linked immuno-sorbent assay (ELISA). As shown in Fig. S3A, 28 clones inhibited the binding of Spike^Wuhan^ with ACE2 by more than 75%. We then examined the neutralizing activity of the 70 clones by using a pseudotyped vesicular stomatitis virus (VSV) encoding a reporter luciferase gene and bearing Spike^Wuhan^ (VSV-SARS-CoV-2^Wuhan^) (Fig. S3B). By comparing the luciferase signal observed in infected cells without antibodies (Fig. S3B; *w/o mAb*, 2.1 × 10^4^ RLU), we selected antibodies that reduced the signal to less than 0.5% as neutralizing antibodies, resulting in the identification of 25 neutralizing clones.

Of the 25 identified clones, CSW1-1805 had strong binding activity against Spike^Wuhan^ (Fig. S2), inhibitory activity against Spike^Wuhan^ and ACE2 binding (Fig. S3A), and neutralizing activity against VSV-SARS-CoV-2^Wuhan^ (Fig. S3B). We then compared the antibody epitope of CSW1-1805 with those of the other neutralizing antibodies. As shown in Fig. S3C, 22 clones inhibited the binding of CSW1-1805 with Spike^Wuhan^, suggesting that they recognized almost the same epitope as CSW1-1805. In contrast, the remaining two clones, CSW2-0611 and CSW2-1353, were less able to inhibit the binding of CSW1-1805 with Spike^Wuhan^, and CSW2-1353 particularly appeared to recognize a different epitope from CSW1-1805 (Fig. S3C, *red bar*). Therefore, we decided to characterize CSW1-1805 and CSW2-1353 in detail.

### Characterization of CSW1-1805 and CSW2-1353 *in vitro* and *in vivo*

Shortly after the first appearance of SARS-CoV-2 in Wuhan, the B.1 lineage with the D614G substitution in the spike protein emerged in Europe and replaced the original Wuhan strain (26). Since the spike protein with the D614G substitution (Spike^B.1^) is reported to be more stable than Spike^Wuhan^ (27), we used Spike^B.1^ in our experiments.

**Fig 1:**
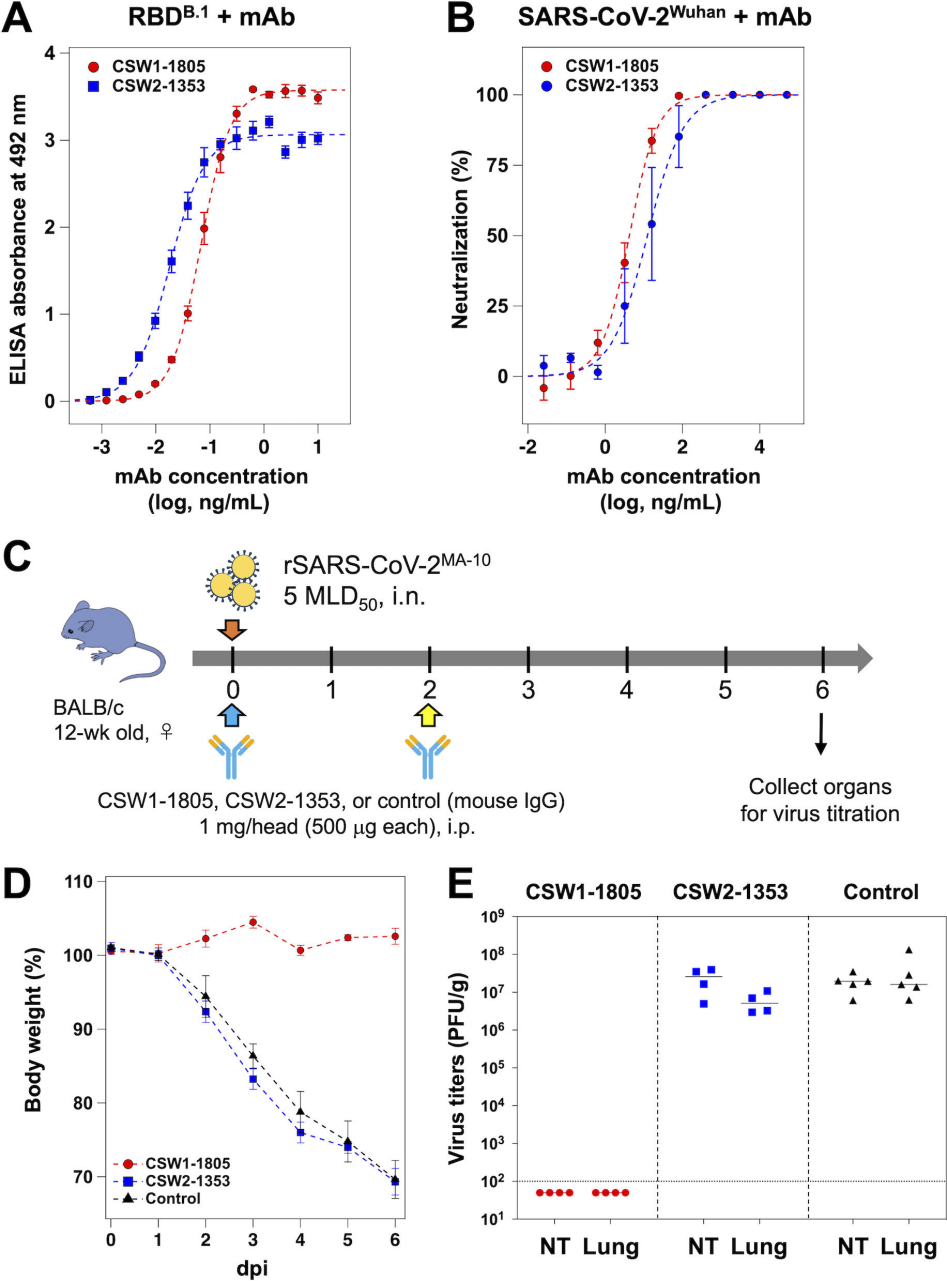
Characterization of the neutralizing antibodies against authentic SARS-CoV-2. (**A**) Binding curves of CSW1-1805 and CSW2-1353 against RBD^B.1^ were obtained by using an ELISA. Assays were performed independently three times (means ± SD). The plots were fitted with a sigmoidal function by using Igor Pro (ver 8.04, Wavemetrics). (**B**) Concentration-dependency of CSW1-1805 and CSW2-1353 for plaque reduction of the SARS-CoV-2 Wuhan strain. Assays were performed independently three times (means ± SD). The plots are expressed as a decreasing percentage of the plaque numbers relative to the control (without antibody) and fitted with a sigmoidal function by using Igor Pro (ver 8.04, Wavemetrics). (**C**) Overview of the animal challenge study. Mice were intranasally infected with 5 MLD_50_ of rSARS-CoV-2MA-10 immediately after intraperitoneally administrating 500 µg of CSW1-1805 or isotype control. Two days later, antibodies were administered for a second time, and 6 days later, organs were collected for virus titration. (**D**) Body weight changes and (**E**) viral titers of nasal turbinate (NT) and lung samples from mice administrated CSW1-1805 (*red*), CSW2-1353 (*blue*), or isotype control (*black*). The dotted line in (**E**) indicates the detection limit.

First, we examined the binding ability of CSW1-1805 and CSW2-1353, which were purified from ascites fluid collected from immunized mice, with the RBD of Spike^B.1^ (Fig. 1A). Although affinity values between IgG and an antigen obtained by using an ELISA (28) are apparent affinities, such values provide useful information for comparing the binding ability of antibodies. The apparent dissociation constant (*K*_d, app_) values between RBD^B.1^ and the antibodies were 4.53 × 10^−10^ M for CSW1-1805 and 1.18 × 10^−10^ M for CSW2-1353 (Fig. 1A; Table S1). Next, to evaluate the neutralizing activity of CSW1-1805 and CSW2-1353 against SARS-CoV-2, we performed a plaque reduction assay using the authentic SARS-CoV-2 Wuhan strain (SARS-CoV-2^Wuhan^) (Fig. 1B). The 50% plaque reduction neutralization test (PRNT50) values calculated from the neutralization curve were 4.05 ng/mL for CSW1-1805 and 14.1 ng/mL for CSW2-1353 (Table S2).

We next evaluated the protective efficacy of CSW1-1805 and CSW2-1353 against SARS-CoV-2 infection in a mouse model. We first generated a recombinant SARS-CoV-2 (rSARS-CoV-2^MA-10^) possessing seven mouse-adapting substitutions (i.e., T285I in nsp4; K2R in nsp7; E23G in nsp8; Q493K, Q498Y, and P499T in the spike; and F7S in orf6) identified in a previous study (29) by using a recently established reverse genetics system (30). Twelve-week-old BALB/c mice were intraperitoneally administered 500 µg of CSW1-1805, CSE2-1353, or mouse IgG as a control and then inoculated intranasally with 5 MLD_50_ of rSARS-CoV-2^MA-10^. Two days later, the mice were intraperitoneally given the same dose of CSW1-1805, CSE2-1353, or mouse IgG (Fig. 1C). Body weights were measured daily for 6 days after infection, and nasal turbinate and lung samples were collected at 6 days post-infection (dpi) for virus titration. There were no clinical signs such as weight loss in the CSW1-1805-treated group, whereas, in the CSW2-1353 and control groups, continuous weight loss was observed starting at 2 dpi, and by 6 dpi, all of the mice in this group had died (Fig. 1D). In the control group, the mean virus titers (±SD) in the nasal turbinates and lungs were 7.23 ± 0.25 log_10_PFU/g and 7.34 ± 0.44 log_10_PFU/g, respectively (Fig. 1E). No virus was detected in either the nasal turbinates or lungs of the CSW1-1805-treated mice, whereas the mean virus titers (±SD) in the CSW2-1353-treated group were 7.26 ± 0.36 log_10_PFU/g in the nasal turbinates and 6.71 ± 0.23 log_10_PFU/g in the lungs (Fig. 1E). These results indicate that CSW1-1805 completely protects mice from rSARS-CoV-2^MA-10^ infection, but CSW2-1353 does not.

### Effect of mutations in the SARS-CoV-2 spike on the reactivity of the neutralizing antibodies

**Fig 2:**
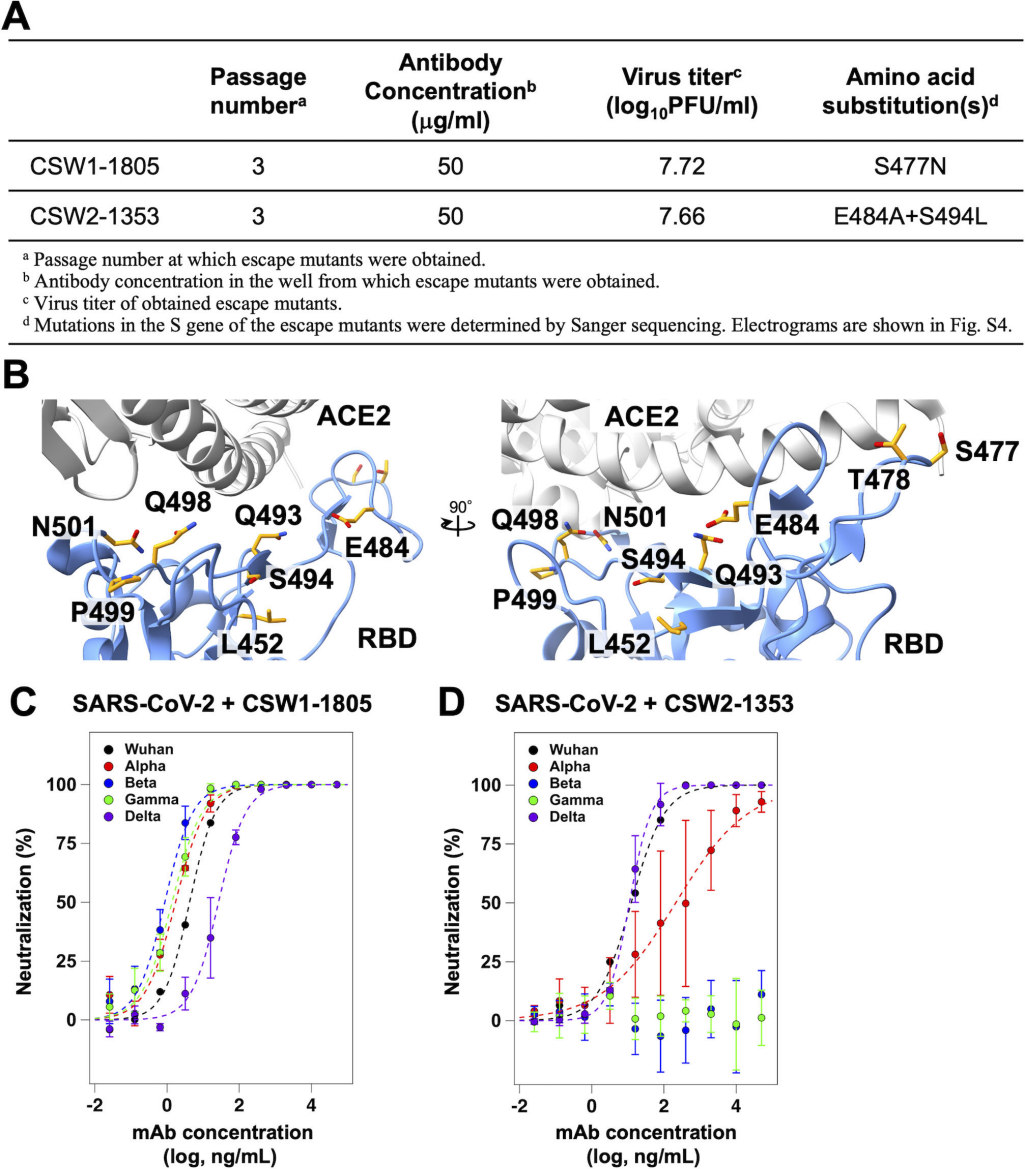
Reactivity of the neutralizing antibodies against SARS-CoV-2 variants. (**A**) Summary of the obtained escape mutant viruses. (**B**) The positions of the mutated residues are shown as sticks in the structure of the RBD-ACE2 complex (PDB ID: 6m0j). Substituted amino acids in rSARS-CoV-2MA-10 and variants located near the antibody-binding interface are also shown as sticks. (**C, D**) Concentration-dependency of (**C**) CSW1-1805 and (**D**) CSW2-1353 for plaque reduction of SARS-CoV-2 variants. Assays were performed independently three times (means ± SD). The plots are expressed as a decreasing percentage of the plaque numbers relative to the control (without antibody) and fitted with a sigmoidal function by using Igor Pro (ver 8.04, Wavemetrics). The results for the SARS-CoV-2 Wuhan strain described in Fig. 1C are also presented for comparison.

To define the epitope of each antibody, we attempted to generate viral escape mutants by repeating virus passages in the presence of each antibody. No mutant was detected after three passages when SARS-CoV-2^Wuhan^ was inoculated at a low multiplicity of infection (MOI) (MOI = 0.1). In contrast, when inoculated at a high MOI (MOI = 2), escape mutant viruses carrying the S477N and E484A/S494L substitutions in their spike protein emerged after three passages in the presence of CSW1-1805 and CSW2-1353, respectively (Fig. 2A; Fig. S4). We also confirmed that CSW1-1805 and CSW2-1353, even at the maximum concentration tested (i.e., 50 µg/mL), failed to neutralize the S477N and E484A/S494L mutants, respectively. These results suggest that S477 and E484/S494 are key residues in the epitopes of CSW1-1805 and CSW2-1353, respectively. In our animal challenge study, CSW2-1353 failed to neutralize rSARS-CoV-2^MA-10^, which possessed Q493K, Q498Y, and P499T mutations in the spike (Fig. 1D and E). The loss of neutralizing activity of CSW2-1353 against SARS-CoV-2^MA-10^ may be due to the Q493K and/or Q498Y mutation, located near S494 (Fig. 2B).

The global spread of SARS-CoV-2 has led to the emergence of several variants, such as Alpha, Beta, Gamma, Delta, and Omicron, which are designated as “variants of concerns (VOCs)” by the World Health Organization (WHO) (31). VOCs have one or more RBD mutation(s) that result in improved binding affinity for ACE2 (32–34) and, in some cases, resistance to neutralization by antibodies (33–37). We, therefore, examined whether CSW1-1805 and CSW2-1353 are effective against SARS-CoV-2 variants to identify key residues of the RBD recognized by the antibodies. The *K*_d, app_ values of CSW1-1805 against the RBD showed that CSW1-1805 retained high affinity for all VOCs tested (Alpha, 4.52 × 10^−10^ M; Beta, 4.82 × 10^−10^ M; Gamma, 4.94 × 10^−10^ M; and Delta, 2.62 × 10^−10^ M), except for Omicron BA.1 (Fig. S5A; Table S1). The loss of CSW1-1805 neutralizing activity against Omicron BA.1 is consistent with the Omicron BA.1 possessing the S477N mutation in the spike protein (Fig. S5C), which was found in the escape mutants from CSW1-1805 (Fig. 2A). In contrast, CSW2-1353 retained high affinity for the Alpha and Delta variants (Alpha, 1.52 × 10^−10^ M; Delta, 1.14 × 10^−10^ M) but lost the ability to bind to the Beta, Gamma, and Omicron BA.1 variants (Fig. S5B; Table S1). Because CSW2-1353 failed to neutralize the escape mutant possessing the E484A mutation in the spike (Fig. 2A), the substitution at E484 in the Beta, Gamma, and Omicron BA.1 variants (Fig. S5C) likely caused the loss of binding ability of CSW2-1353 to these variants.

Next, we evaluated the neutralizing activity of CSW1-1805 and CSW2-1353 against authentic SARS-CoV-2 variants. Because neither CSW1-1805 nor CSW2-1353 bound to the RBD of the Omicron variant (Fig. S5A and B), we tested antibody neutralization of previous VOCs from Alpha to Delta. The PRNT50 values of CSW1-1805 showed higher neutralizing activity against the Alpha variant (1.89 ng/mL), Beta variant (1.00 ng/mL), and Gamma variant (1.80 ng/mL) and 6.5-fold lower neutralizing activity against the Delta variant (26.4 ng/mL) compared with the Wuhan strain (Fig. 2C; Table S2). The Delta variant possesses the L452R and T478K mutations in the RBD region (Fig. S5C). Given that the neutralizing activity of CSW1-1805 was lost by the substitution of Ser for Asn at position 477, which is next to T478 (Fig. 2B), the T478K substitution was the likely cause of the reduced neutralizing activity of CSW1-1805 against the Delta variant. In contrast, the PRNT50 values of CSW2-1353 showed high neutralizing activity against the Delta variant (10.9 ng/mL) but significantly lower neutralizing activity against the Alpha variant (200 ng/mL) and, as expected from the ELISA data (Fig. S5B), no neutralizing activity against the Beta or Gamma variants (Fig. 2D; Table S2). In summary, CSW1-1805 neutralized several strains (i.e., Alpha, Beta, Gamma, and Delta), whereas CSW2-1353 neutralized Alpha and Delta but lost activity against Beta and Gamma, indicating that CSW1-1805 and CSW2-1353 recognize different epitopes.

### Epitopes of CSW1-1805 and CSW2-1353

**Fig 3:**
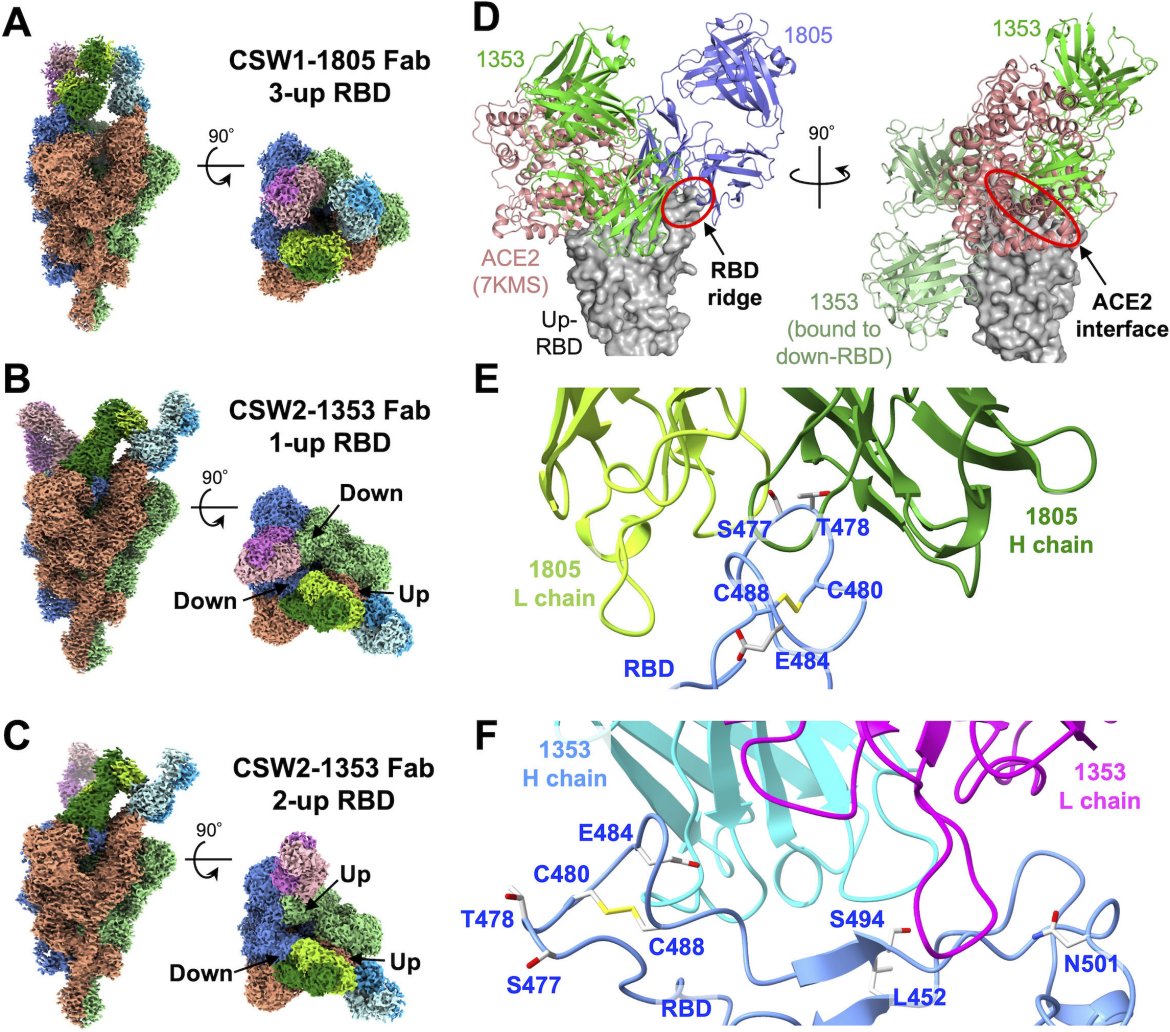
Structural analysis of spike–Fab complexes. (**A–C**) Final sharpened maps of (**A**) Spike^B.1^ + CSW1-1805, (**B**) Spike ^B.1^ + CSW2-1353 (1-up RBD), and (**C**) Spike ^B.1^ + CSW2-1353 (2-up RBD) in two orthogonal views: side view, left panels; and top view, right panels. The map regions corresponding to each protomer in the spike protein are colored in blue, red, and green, respectively. The map regions corresponding to the light and heavy chains of each Fab are colored in light green/cyan/pink and deep green/cyan/pink, respectively. (**D**) Superimposed 3D structures around one up-RBD. ACE2 molecules (from PDB: 7KMS) are also shown. The right panel shows approximately 90^o^-rotated views of the left panel. Neighboring CSW2-1353 and ACE2 molecules are shown only in the right panel. (**E, F**) Close-up views of the interface between (**E**) the up-RBD and CSW1-1805 Fab or (**F**) the down-RBD and CSW2-1353 Fab. The RBD is colored in blue; the heavy and light chains of CSW1-1805 are in dark green and light green, respectively; and the heavy and light chains of CSW1-1353 are in cyan and magenta, respectively. Residues substituted in the Alpha, Beta, Gamma, Delta, and escape mutants found in this study are shown as sticks.

To investigate the binding sites of CSW1-1805 and CSW2-1353, we performed cryo-EM single particle analyses of the Spike^B.1^ in complex with the Fabs of CSW1-1805 and CSW2-1353 (Table S3). Many spike trimer particles were observed in the micrographs (Fig. S6A; Fig. S7A), and the 2D class averages showed various particle orientations (Fig. S6B; Fig. S7B). For CSW1-1805, the Fab bound to all three RBDs of the spike trimer, and all three RBDs were in the up conformation (3-up RBD) (Fig. 3A; Fig. S6C). The overall map resolution reached 3.62 Å (Fig. S6D). For CSW2-1353, there were two conformations: down-up-down (1-up RBD) and down-up-up (2-up RBD) in a counterclockwise direction (*seen from the top*), and the Fab bound to all three RBDs regardless of the conformation (Fig. 3B and C; Fig. S7C and D). The overall map resolutions of the 1-up RBD and the 2-up RBD reached 3.66 Å and 3.97 Å, respectively (Fig. S7E and F). As we could not build atomic models of CSW1-1805 and CSW2-1353 due to the low local resolution (Fig. S6C; Fig. S7C and D), homology models of CSW1-1805 and CSW2-1353 were generated based on these variable region sequences (Fig. S8) and manually fitted to the maps (Fig. S9A and B). In addition, the Fabs of CSW2-1353, which bound to the down RBD and to the counterclockwise adjacent up RBD, interacted with each other (Fig. S9B); the up RBD was shifted outward compared with the up RBD bound to the Fab of CSW1-1805 (Fig. S9C). In other words, the interaction between the Fabs of CSW2-1353 attached to the RBDs may have stabilized the conformation of the adjacent pair of RBDs.

CSW1-1805 bound to the loop region (Y473–Y489) at the RBD ridge (Fig. 3D and E). The cryo-EM structure of the CSW1-1805 Fab in complex with Spike^B.1^ showed that CDR L3 and H3 of CSW1-1805 surrounded S477 and T478 of the RBD ridge, respectively (Fig. S9D and E). Based on the binding assay and viral escape mutation study (Fig. S5A; Fig. 2A), S477 makes an essential binding contribution. In contrast, T478 is part of the binding site but may not be important for binding because T478K mutations did not have much impact on binding affinity or neutralizing activity (Fig. S5A; Fig. 2C). Although this loop region also contains E484, CSW1-1805 neutralized the Beta and Gamma variants (Fig. 2C), which possess the E484K mutation (Fig. S5C). Given that the E484 sidechain faces away from the contact site of CDR L1 and H3 with the RBD ridge (Fig. S9F), E484 does not appear to be essential for the interaction with CSW1-1805.

CSW2-1353 recognized a wide area on the top of the RBD head, which overlapped extensively with the ACE2-RBD interaction interface (Fig. 3D and F). E484 of the RBD participated in the interactions with CDR H2 of CSW2-1353 (Fig. S9G). This is consistent with the complete loss of binding and neutralizing activity against the Beta and Gamma variants (Fig. S5B; Fig. 2D), which possess the E484K mutation (Fig. S5C). L452, Q493, and S494 were located between CDR H3 and L1 (Fig. S9H and I), and Q498 and N501 were positioned at the outer rim of CDR L1 (Fig. S9J). CSW2-1353 lost neutralizing activity against SARS-CoV-2^MA-10^, which possessed the Q493K and Q498Y mutations (Fig. 1D and E) and the escape mutant with the E484A and S494L mutations (Fig. 2A), suggesting that Q493, S494, and Q498 contact CSW2-1353. In contrast, L452 and N501 may contribute to the interaction but are not essential because CSW2-1353 retained neutralizing activity against Alpha and Delta variants possessing N501Y and L452R mutations, respectively (Fig. 2D).

### Binding properties of the ridge-binding antibody CSW1-1805

**Fig 4:**
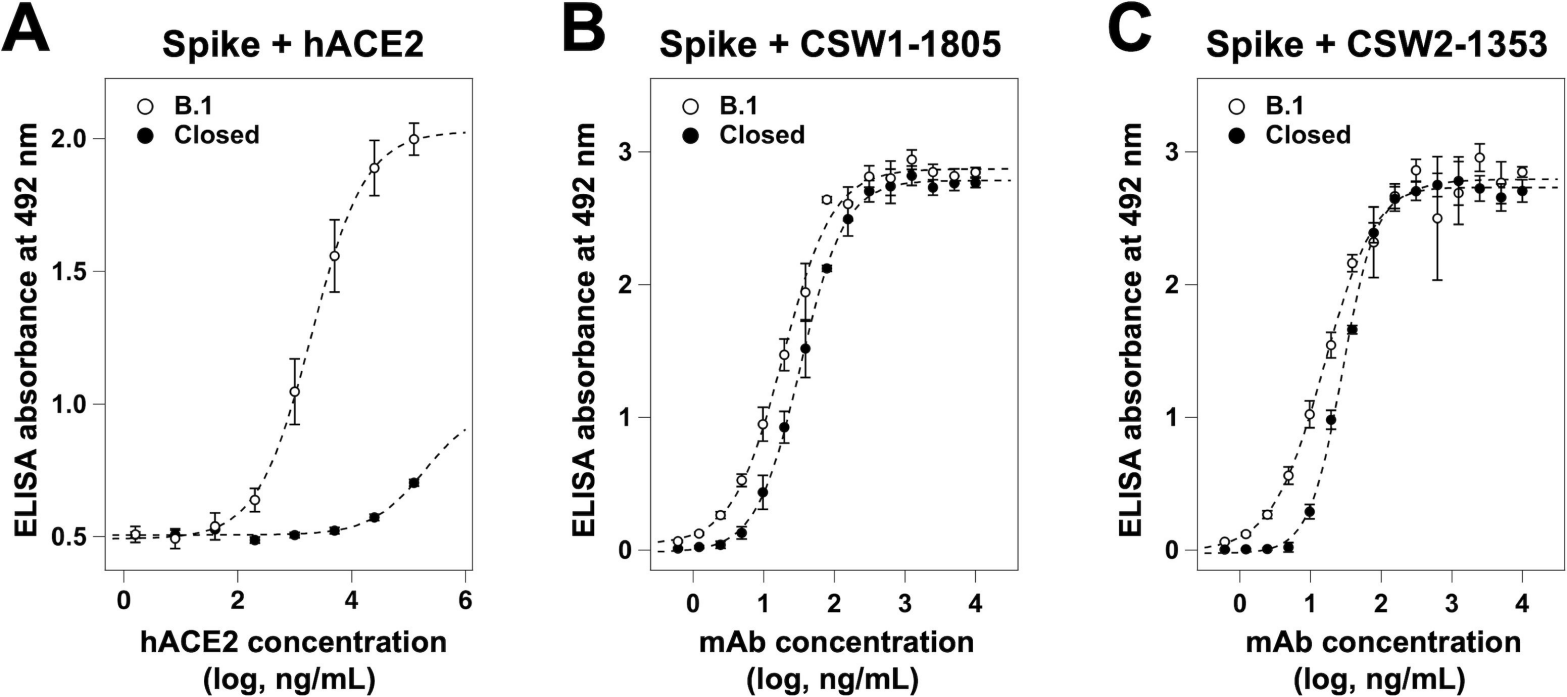
Binding analysis of the neutralizing antibodies to the down-RBD conformation. Binding curves of (**A**) hACE2-His-Strep, (**B**) CSW1-1805, and (**C**) CSW2-1353 to Spike^B.1^ (*open circle*) and Spike^closed^ (*closed circle*) were obtained by using an ELISA. Assays were performed independently three times (means ± SD). The plots were fitted with a sigmoidal function by using Igor Pro (ver 8.04, Wavemetrics).

While the CSW2-1353 Fab/Spike^B.1^ complex had a mixture of up and down RBD conformations (Fig. 3B and C), the CSW1-1805 Fab/Spike^B.1^ complex had only the 3-up RBD conformation (Fig. 3A). These results suggest that CSW2-1353 recognizes both the up and down RBD conformations, whereas CSW1-1805 may specifically recognizes the up RBD conformation. To explore this possibility, we examined whether the antibodies could bind to a mutant spike protein (Spike^B.1^ with the S383C/D985C mutations, named Spike^closed^) (38–40) in which all of the RBDs were stabilized in the down conformation, or “closed state.” Closed state spike proteins have shown reduced binding affinity with ACE2 and infectivity of pseudo-typed SARS-CoV-2 (40, 41). We verified the reduced binding affinity of purified Spike^closed^ with ACE2, compared to Spike^B.1^, by ELISA (Fig. 4A). Then, we examined the binding of CSW1-1805 and CSW2-1353 to Spike^closed^ by ELISA; contrary to our expectations, both antibodies bound to the Spike^closed^ with similar affinity to Spike^B.1^ (Fig. 4B and C). Hence, the loop region, the epitope of CSW1-1805, was accessible in both the up and down RBD conformations.

Even though CSW1-1805 also bound to the down RBD conformation (Fig. 4B), Spike^B.1^ in complex with CSW1-1805 eventually formed only the up RBD conformation (Fig. 3A), unlike CSW2-1353 (Fig. 3B and C). The loop region of the RBD harbors an intramolecular disulfide bond between Cys480 and Cys488 (Fig. 3E). We performed a cryo-EM analysis of the spike protein with the C480A mutation (Spike^C480A^) (Fig. S10A and B) because the flexibility of the loop region should be increased due to the cleavage of the disulfide bond. The structure of Spike^C480A^ showed only the 2-up RBD conformation (Fig. S10C), whereas that of Spike^B.1^ showed only the 1-up RBD conformation from our previous analysis (42). The overall map resolution reached 3.04 Å (Fig. S10D). Although we could not trace the main chain in the loop region (Fig. S11), these results suggest that the increased flexibility of the loop region of the RBD may weaken interactions with an adjacent protomer and subsequently induce a transition to the up RBD conformation. Perturbations to the loop region, such as antibody binding, may also affect interactions that stabilize the down RBD conformation and induce a transition to the up RBD conformation.

**Fig 5:**
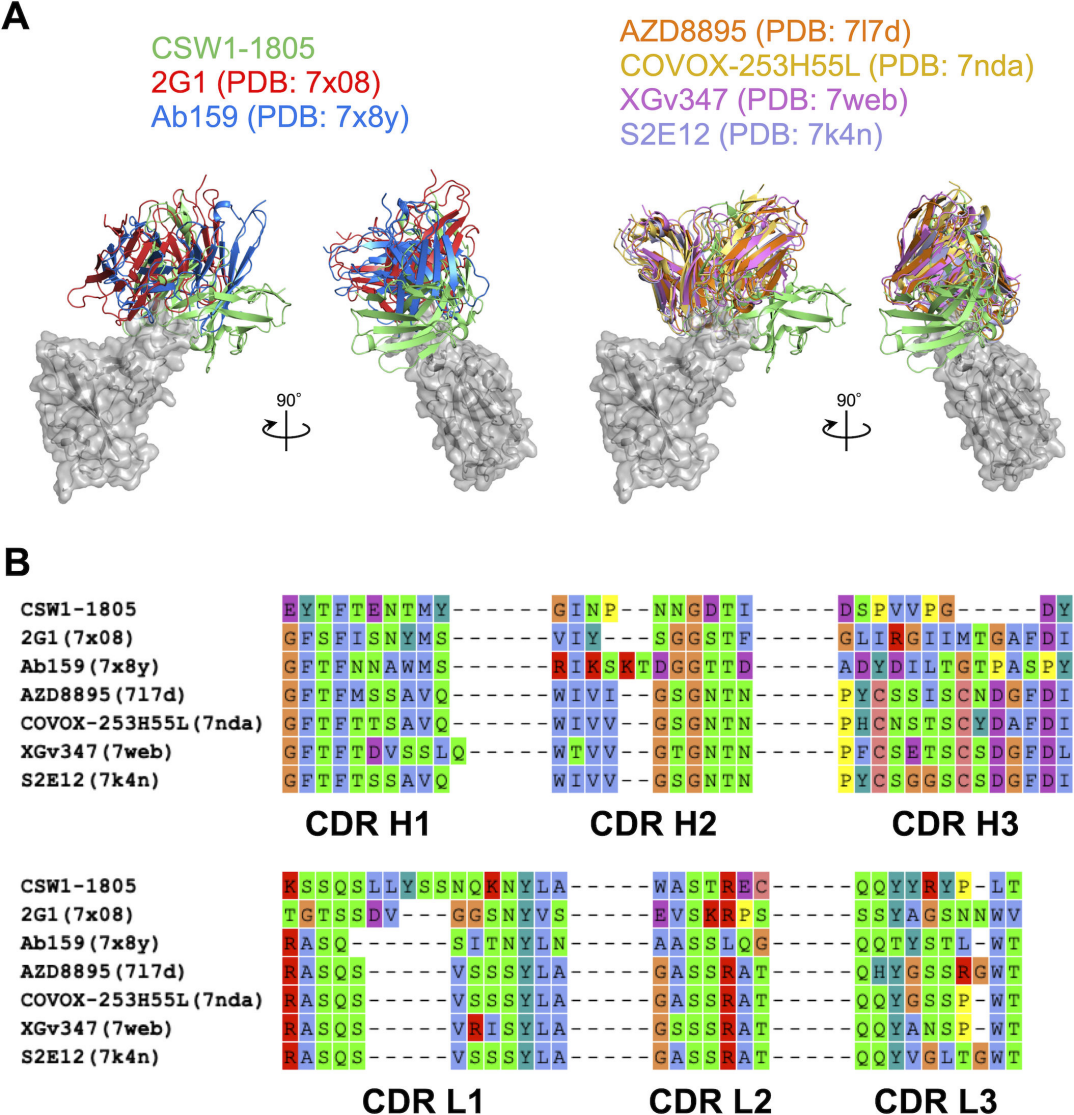
Comparison of neutralizing antibodies that bind to the RBD ridge. (**A**) Superimposed structures of CSW1-1805, 2G1, and Ab159 on the RBD in *the left panel* and CSW1-1805, AZD8895, COVOX-253H55L, XGv347, and S2E12 on the RBD in *the right panel* are shown in two orthogonal views. (**B**) Alignment of the amino acid sequences of the CDRs within the heavy (*upper panel*) and light (*lower panel*) chain variable domains are shown. Amino acid are colored according to the Clustal color scheme (Hydrophobic amino acids: A, I, L, M, F, W, V in blue; positively charged amino acids: K, R in red; negatively charged amino acids: E, D in magenta; polar amino acids: N, Q, S, T in green; C in pink; G in orange; P in yellow; aromatic amino acids: H, Y in cyan).

Cryo-EM structures showed that CSW2-1353 had an epitope that broadly overlapped the ACE2-bound interface of the RBD, whereas CSW1-1805 recognizes the narrow loop region at the RBD ridge adjacent to the ACE2-RBD interaction interface (Fig. 3D). Despite its relatively narrow epitope, CSW1-1805 had an affinity comparable to that of CSW2-1353, which has a broad epitope region (Table S1) and could completely protect against SARS-CoV-2 infection in the mouse model (Fig. 1C through E). Several antibodies that bind to the ridge of the RBD have been reported (43–48). Therefore, we compared CSW1-1805 with six previously reported antibodies. Structural alignment showed that all antibodies, including CSW1-1805, bind to the ridge-centered epitope, but CSW1-1805 exhibits a binding direction distinct from the other antibodies (Fig. 5A). We further aligned the CDR sequences of each antibody. For AZD8895, COVOX-253H55L, XGv347, and S2E12, which had similar binding modes (Fig. 5A), each CDR had similar characteristics in terms of length and amino acid properties (Fig. 5B). Antibodies 2G1, Ab159, and CSW1-1805 had different CDR lengths and amino acid properties from each other; in particular, CDR H3 and L1 of CSW1-1805 were distinctly different from those of the other antibodies (Fig. 5B), and these regions significantly contributed to the interaction with the loop in the RBD (Fig. S9F). These findings suggest that CSW1-1805 has different binding characteristics than those of previously reported antibodies.

## DISCUSSION

The interaction between the spike RBD and ACE2 is essential for SARS-CoV-2 infection (5–8). Therefore, numerous antibodies that recognize the RBD have been extensively identified and characterized with the aim of developing antibody drugs effective against COVID-19 (20, 21). In this study, we screened mouse monoclonal antibodies for neutralizing activity against SARS-CoV-2 and found CSW1-1805, a monoclonal antibody that exhibited high neutralizing activity against several variants (i.e., Alpha, Beta, Gamma, and Delta variants) *in vitro* (Fig. 2C) and provided complete protection against SARS-CoV-2 infection in a mouse model (Fig. 1C through E). Our cryo-EM and biochemical analyses revealed that CSW1-1805 binds to a narrow loop region at the RBD ridge in both conformations and lock the RBD in the up conformation (Fig. 3E; Fig. 4B). Furthermore, comparisons of CSW1-1805 with previously reported antibodies that bind to the RBD revealed that its epitope and CDR sequences differ from those of other antibodies (Fig. 5), suggesting that CSW1-1805 has unique binding properties.

Antibodies targeting the RBD can be divided into several categories based on their binding features, and various classification systems have been proposed: Hastie et al. (22) and Piccoli et al. (23) classified the RBD-directed antibodies into seven and six groups, respectively, based on their binding epitope in the RBD, whereas Barnes et al. (24) categorized them into four groups based on their binding mode to the spike protein (22–24). The epitope of CSW2-1353 overlaps extensively with the outer edge of the ACE2-binding site (Fig. 3D), which corresponds to the epitope termed “site Ib” by Hastie et al. (22) and “RBD-4” by Piccoli et al. (23). In addition, CSW2-1353 could bind in either the up or the down RBD conformation (Fig. 3B and C). This binding property is comparable to that of “Class 2” antibodies defined by Barnes et al. (24), indicating that CSW2-1353 should be included in the previously characterized neutralizing antibodies targeting the ACE2-binding site. CSW1-1805 also shares the “Class 2” antibody feature of binding to both up and down RBDs (Fig. 3A; Fig. 4B); however, the binding epitope of CSW1-1805 is the narrow loop region at the RBD ridge adjacent to the ACE2-binding site (Fig. 3D). This differs from the binding mode of “Class 2” antibodies reported by Barnes et al. (24) and is closer to that of tixagevimab, a “Class 1” antibody (Fig. 5A). Although this binding region partially covers the epitope that spans the ACE2-binding site termed “site Ib” by Hastie et al. (22) and “RBD-1 and -2” by Piccoli et al. (23), the epitope of CSW1-1805 appears to be different from those previously defined. Therefore, we suggest that although CSW1-1805 is a “Class 2” antibody, it may have additional properties.

Although the epitope of CSW1-1805 appears to be narrow compared with that of other RBD-targeting neutralizing antibodies (20–24), CSW1-1805 showed high neutralizing activity *in vitro* (Fig. 1B) and completely protected mice from SARS-CoV-2 infection (Fig. 1D and E). One reason for this strong activity may be that CSW1-1805 binds to both the up and down RBD conformations (Fig. 3A; Fig. 4B). The down RBD conformation is disadvantageous for binding to ACE2 because the ACE2 interface is buried in the down RBD conformation (9, 10, 26); however, it is advantageous for immune evasion because of its reduced accessibility to neutralizing antibodies that recognize the ACE2 interface (40, 49, 50). Structural analyses have shown that the SARS-CoV-2 spike possesses a higher proportion of the down RBD conformation than SARS-CoV (6, 9, 10, 51, 52), suggesting that SARS-CoV-2 may reduce the accessibility of neutralizing antibodies to its RBD by using the down RBD conformation predominantly, resulting in increased efficiency of immune evasion (49). The ability to bind to both the up and down RBD conformations is, therefore, an important consideration in the development of useful neutralizing antibodies.

We also found that the binding of CSW1-1805 stabilized the RBD in the up conformation (Fig. 3A). Several other antibodies targeting the RBD ridge have been reported (Fig. 5) (43–48) including tixagevimab (AZD8895) (45), which was previously authorized in many countries as a prophylactic for COVID-19. Cryo-EM analysis has revealed that S2E12 forms its spike-antibody complex with 3-up RBD since the epitope of the S2E12 antibody is inaccessible in the down RBD conformation (48). Despite being able to bind strongly to both the up- and down RBD (Fig. 4B), CSW1-1805 does not form a mixture of down and up RBD, like COVOX-253H55L (46) but, like S2E12, forms only 3-up RBDs (Fig. 3A). This suggests that CSW1-1805 has the unique ability to induce a conformational change in the RBD from down to up after binding. The CDR of CSW1-1805, especially H3 and L1, has different characteristics from other ridge-binding antibodies such as COVOX-253H55L (Fig. 5), which may explain the unique binding property of CSW1-1805: that is, its ability to bind a narrow loop region at the ridge induces the RBD to adopt the up conformation. Indeed, the preferred RBD conformation is in the up-state with perturbation of the loop region, which is the epitope of CSW1-1805 (Fig. S10; Fig. S11). Previous studies have proposed that ACE2 binds specifically to the up RBD conformation rather than the down RBD conformation during SARS-CoV-2 infection (26, 49, 53). Therefore, elucidating the mechanisms that regulate the up and down conformations of the RBD is a key issue, and a detailed examination of the RBD ridge may reveal new insights.

On 5 May 2023, the WHO announced the end of the “public health emergency of international concern” with respect to COVID-19, but a global epidemic is still ongoing. While research and development of antibody drugs have progressed, SARS-CoV-2 has also evolved and acquired immune-escape capability. The neutralizing activity of approved antibody drugs against the currently circulating Omicron variants has been significantly reduced (54). Monotherapy with antibody drugs increases the risk of new escape variants (55–57); therefore, new antibody drugs are needed. Although, unfortunately, the neutralizing activity of CSW1-1805 against the Omicron variant was lost due to the S477N substitution, we hope that our characterization of antibodies targeting the RBD ridge including CSW1-1805 will provide valuable information for future neutralizing antibody development.

## MATERIALS AND METHODS

### Cells

The Expi293F cells (Thermo) were maintained in the HE400AZ medium (Gmep Inc., Japan) at 37°C in 8% CO_2_. Transmembrane protease, serine 2 (TMPRSS2)-expressing Vero E6 (VeroE6/TMPRSS2) cells (58) were obtained from the Japanese Collection of Research Bioresources Cell Bank (JCRB1819) and maintained in high-glucose Dulbecco’s modified Eagle medium (DMEM) (nacalai tesque, Japan) containing 10% fetal bovine serum (FBS) (Sigma), 100 units/mL penicillin (nacalai tesque), 100 µg/mL streptomycin (nacalai tesque), and 1 mg/mL G418 (nacalai tesque) at 37°C in 5% CO_2_. The LentiX-293T cells (Clontech) were maintained in DMEM containing 10% FBS, 100 units/mL penicillin (nacalai tesque), and 100 µg/mL streptomycin (nacalai tesque) at 37°C in 5% CO_2_.

### Viruses

A Wuhan strain (strain SARS-CoV-2/Hu/DP/Kng/19-020) was kindly provided by Dr. Sakuragi at the Kanagawa Prefectural Institute of Public Health. An Alpha variant (B.1.1.7 lineage; strain hCoV-19/Japan/QHN001/2021), a Beta variant (B.1.351 lineage; strain hCoV-19/Japan/TY8-612/2021), and a Gamma variant (P.1 lineage; strain hCoV-19/Japan/TY7-503/2021) were obtained from the National Institute of Infectious Diseases, Japan. A Delta variant (B.1.617.2; strain BKVC-127) was isolated at the Research Foundation for Microbial Diseases of Osaka University (BIKEN), Japan. Previously, seven amino acid mutations (T285I in nsp4; K2R in nsp7; E23G in nsp8; Q493K, Q498Y, and P499T in the spike; F7S in orf6) have been identified in the mouse-adapted SARS-CoV-2 strain (SARS-CoV-2 MA-10) (29). In this study, SARS-CoV-2 with these mutations was generated as a recombinant mouse-adapted SARS-CoV-2 (rSARS-CoV-2^MA-10^) by using our established reverse genetics method (30).

All viruses were propagated in VeroE6/TMPRSS2 cells in DMEM (nacalai tesque) containing 2% FBS (Sigma), 100 units/mL penicillin (nacalai tesque), 100 µg/mL streptomycin (nacalai tesque), and 1 mg/mL G418 (nacalai tesque) at 37°C in 5% CO_2_. The virus stocks were stored at −80°C until use. The infectious virus titer of SARS-CoV-2 isolates and rSARS-CoV-2^MA-10^ was determined as plaque-forming units (PFU) by use of a plaque assay and the 50% tissue culture infective doses (TCID_50_), respectively, as described previously (30, 59).

### Plasmid construction

The codon-optimized gene of the SARS-CoV-2 spike protein (Strain Wuhan-Hu-1, GenBank: QHD43416) and human angiotensin-converting enzyme 2 (ACE2; GenBank: NM_021804) was designed for expression in mammalian cells and synthesized from GeneArt DNA Synthesis (Thermo). For the expression of the recombinant spike protein, the sequence encoding the spike ectodomain (a.a. residue 1–1,208) with proline substitutions at residues 986 and 987, a “GSAS” substitution at the furin cleavage site (a.a. residues 682–685), and the C-terminal foldon trimerization motif followed by an octa-histidine tag (Fig. S1A) was cloned into a pcDNA3.1 expression vector (Invitrogen) (designated as pcDNA3.1/SARS-CoV-2_Spike). Amino acid mutations identified in the spike protein of the B.1 lineage (D614G), B.1.1.7 lineage (Alpha variant: del69-70, del144, N501Y, A570D, D614G, P681H, T716I, S982A, D1118H), B.1.351 lineage (Beta variant: D80A, D215G, K417N, E484K, N501Y, D614G, A701V), P.1 lineage (Gamma variant: L18F, T20N, P26S, D138Y, R190S, K417T, E484K, N501Y, D614G, H655Y, T1027I), B.1.617.2 lineage (Delta variant: T19R, del157-158, L452R, T478K, D614G, P681R, D950N), and B.1.1.529 lineage (Omicron variant BA.1: A67V, del69-70, T95I, del142-144, Y145D, del211, L212I, ins214EPE, G339D, S371L, S373P, S375F, S477N, T478K, E484A, Q493R, G496S, Q498R, N501Y, Y505H, T547K, D614G, H655Y, N679K, P681H, N764K, D796Y, N856K, Q954H, N969K, L981F) were introduced as described previously (60). For the expression of the recombinant S1 proteins, the sequence encoding S1 including the N-terminal native signal peptide (a.a. residue 1–660) followed by a C-terminal octa-histidine tag was cloned into a pcDNA3.1 expression vector (designated as pcDNA3.1/SARS-CoV-2_S1). For the expression of the recombinant NTD proteins, the sequence encoding the NTD including the N-terminal native signal peptide (a.a. residue 1–305) followed by a C-terminal octa-histidine tag was cloned into a pcDNA3.1 expression vector (designated as pcDNA3.1/SARS-CoV-2_NTD). For the expression of the recombinant RBD proteins, the RBD (a.a. residue 319–541) with the IL-2 signal sequence (YRMQLLSCIALSLALVTNS) at the N-terminus and an octa-histidine tag at the C-terminus was cloned into a pcDNA3.1 expression vector (designated as pcDNA3.1/SARS-CoV-2_RBD). For the expression of the recombinant ACE2 proteins, the sequence encoding the NTD including the N-terminal native signal peptide (a.a. residue 1–615) followed by a C-terminal octa-histidine tag and Strep-tag was cloned into a pcDNA3.1 expression vector [designated pcDNA3.1/hACE2(1–615)-His-Strep]. For the generation of the pseudo-typed SARS-CoV-2, the spike protein sequence with a C-terminal 19 amino acid deletion was cloned into the pCAGGS expression vector (61) (designated as pCAG/SARS-CoV-2_S^Δ19^).

### Preparation of protein samples

Expi293f cells (Thermo) were transfected with plasmids expressing the recombinant spike, S1, NTD, RBD, and ACE2 proteins by using the Gxpress 293 Transfection Kit (Gmep Inc.) and following the manufacturer’s protocol. At 5 days post-transfection, culture supernatants were harvested, and His-tagged proteins were purified by Ni-NTA affinity chromatography using Ni Sepharose 6 Fast Flow (Cytiva), followed by size exclusion chromatography using Superdex 200 Increase 10/300 Gl (Cytiva) equilibrated with 50 mM HEPES (pH 7.4) and 200 mM NaCl.

Fab fragments of IgG antibodies and the spike protein with the D614G mutation of the B.1 lineage (Spike^B.1^) complexed with the Fab were prepared as described previously (62) with some modifications. Briefly, for the generation of the Fab fragments, 10 mg/mL IgG in PBS was mixed with 10 mg/mL papain (Wako, Japan) in PBS, 5 mM EDTA, and 10 mM L-cysteine at a 100:5 (IgG:papain) (wt/wt) ratio and then incubated at 37°C for 1 h. Prior to mixing the IgG and papain, the dissolved papain in PBS containing EDTA and L-cysteine was incubated at 37°C for 1 h to activate the papain. Undigested IgG and Fc fragments were removed by using HiTrap MabSelect SuRe (Cytiva) equilibrated with PBS. Cleaved Fabs were further purified by size exclusion chromatography using Superdex 200 Increase 10/300 Gl (Cytiva) equilibrated with 50 mM HEPES (pH 7.4) and 200 mM NaCl. For the preparation of the Spike^B.1^-Fab complexes, purified Fab fragment was mixed with Spike^B.1^ at a 2:1 (Fab:spike monomer) molar ratio and then incubated at 4°C for 1 h. After this incubation, the Spike^B.1^-Fab complexes were purified by size exclusion chromatography using Superdex 200 Increase 10/300 Gl (Cytiva) equilibrated with 50 mM HEPES (pH 7.4) and 200 mM NaCl.

### Monoclonal antibody production and screening

Monoclonal antibodies (mAbs) against the spike protein of the Wuhan strain (Spike^Wuhan^) were produced by Bio Matrix Research Inc. (Japan). Seven-week-old female BALB/c mice (The Jackson Laboratory Japan, Inc., Japan) were first immunized with 10 µg of purified Spike^Wuhan^ conjugated with Freund’s Incomplete Adjuvant containing 30 µg of ODN-1826 (GeneDesign Inc. as Ajinomoto Bio-Pharma Services, Japan) by subcutaneous injection. Immunization was performed three times every 2 weeks using 5 µg of purified Spike^Wuhan^ mixed with Sigma Adjuvant System (SAS) (Sigma). Then, additional intravenous injections (5 µg each) were given two more times at weekly intervals. Three days after the final boost, spleen cells were isolated and fused with the P3-X63-Ag8.653 (63) mouse myeloma cell line in the presence of 50% polyethylene glycol (PEG, MW 3550, Sigma). Cells were seeded in 96-well plates and incubated at 37°C, with 5% CO_2_, 95% humidity. After 7–10 days of culture in RPMI-1640 containing 10% heat-inactivated fetal calf serum (Hy clone, Cytiva), supplemented with hypoxanthine (1 × 10^−4^ M), aminopterin (4 × 10^−7^ M), and thymidine (1.6 × 10^−5^ M; HAT; Sigma), hybridomas that produced an antibody that bound with Spike^Wuhan^ were screened by using an immunoprecipitation method (proprietary technology of Bio Matrix Research Inc.) and an indirect ELISA method. For the indirect ELISA, briefly, 25 ng of Spike^Wuhan^ was coated on an ELISA plate (F96 Maxisorp Nunc Immuno plate, Thermo) and incubated with fivefold diluted hybridoma culture supernatant. Spike-binding mAb was detected by using a mixture of HRP-conjugated anti-mouse IgG antibodies (10,000-fold dilution of goat anti-mouse IgG1, #A90-105P; goat anti-mouse IgG2a, #A90-107P; and goat anti-mouse IgG2b, #A90-109P; Bethyl, USA). Selected hybridomas were cloned by limiting dilution to obtain single clones.

Anti-spike mAbs, CSW1-1805 and CSW2-1353, were purified from mouse ascites fluid by affinity chromatography. Briefly, BALB/c nude mice were primed by intraperitoneal (i.p.) inoculation with pristane for 7 days, and then hybridoma cells were administered by i.p. injection. Ten days post-administration of the hybridoma cells, the ascites fluid was collected. The antibodies in the ascites fluid were purified by using Protein A affinity matrix UNOshere (Bio-Rad). The variable regions of CSW1-1805 and CSW2-1353 were sequenced by Bio-Peak Inc. (Gunma, Japan) using hybridoma cells. The CDR of the antibodies was identified by using the AbM definition method (64).

The animal experiments were approved by the Ethics Committee of Laboratory Animal Experiment of Biomatrix Research Inc. (No. 2019-02), and all experiments were carried out according to the guidelines of this committee.

### Enzyme-linked immuno-sorbent assay

For the analysis of anti-spike mAb binding to SARS-CoV-2-related proteins, spike proteins (50 ng) dissolved in 100 mM carbonate/bicarbonate buffer at pH 9.6 were coated on 96-well plates (Nunc-Immuno Plate CII, Thermo) overnight at 4°C. After three washes with PBS containing 0.1% (vol/vol) Tween 20 (PBS-T), the plates were blocked with PBS containing 5 mg/mL BSA overnight at 4°C. After three washes with PBS-T, purified anti-spike antibodies or culture supernatants of hybridomas secreting anti-spike mAb were diluted in PBS, added to each well, and incubated for 2 h at room temperature. To detect spike-binding antibodies, HRP-conjugated anti-mouse IgG antibody (10,000-fold dilution, #115-035-003, Jackson Immuno Research) diluted in PBS was added to each well and incubated for 2 h at room temperature. *O*-phenylenediamine and 0.1% H_2_O_2_ in 100 mM sodium citrate buffer at pH 5.0 were used as the substrate solution. The absorbance was read at 492 nm using a plate reader (Multiskan FC, Thermo).

To test mAb inhibition of ACE2 binding with the spike, Spike^Wuhan^ was coated on the plates, treated with culture supernatants of hybridomas secreting anti-spike mAbs or medium as a control, and then incubated with 5 ng of biotinylated ACE2 (#10108-H08-B, Sino Biological). Spike^Wuhan^-binding biotinylated ACE2 was detected by using HRP-conjugated Streptavidin (5,000-fold dilution, #SA-5004, VECTOR, USA).

Antibody epitope comparisons were performed by using a biotinylated anti-spike mAb, CSW1-1805. To obtain biotinylated CSW1-1805, affinity-purified IgG fractions from culture supernatants of CSW1-1805-secreting hybridomas were treated with EZ-Link Sulfo-NHS-LC-Biotin (Thermo) according to the manufacturer’s instructions. Biotinylated CSW1-1805 was added to each well after treatment of the well-coated Spike^Wuhan^ with culture supernatants of mAb-secreting hybridomas or medium as a control. Spike^Wuhan^-binding biotinylated CSW1-1805 was detected by using HRP-conjugated Streptavidin (5,000-fold dilution, #SA-5004, VECTOR, USA).

To examine the binding of hACE2 with the spike, plates were coated with Spike^B.1^ or Spike^Closed^, blocked with BSA, and then incubated with serially diluted hACE2-His-Strep in PBS for 2 h at room temperature. After three washes with PBS-T, hACE2-His-Strep bound to the spike proteins was detected by using an anti-Strep-tag II antibody (1,000-fold dilution, #ab76949, Abcam) as the primary antibody and HRP-conjugated anti-rabbit IgG antibody (10,000-fold dilution, #111-035-003, Jackson Immuno Research) as the secondary antibody.

### Pseudotyped SARS-CoV-2 neutralization assay

The VSV variant NC12.1, derived from the Indiana strain, was provided by M. Whitt. Pseudotyped VSV bearing the SARS-CoV-2 spike protein was generated as previously described (65) with some modifications. Briefly, about 80% confluent LentiX-293T cells in a collagen-coated tissue culture dish were transfected with the SARS-CoV-2 S expression vector pCAG/SARS-CoV-2_S^Δ19^. After 24 h of incubation, the transfected cells were infected with G-complemented VSVΔG harboring the luciferase gene in place of the G gene (66). At 24 h post-infection, the culture supernatants containing pseudotyped VSVs bearing the SARS-CoV-2 spike protein (VSV-SARS-CoV-2) were collected by centrifugation and then stored at −80°C until use.

To examine the neutralization activity of antibodies against the pseudotyped viruses, 10-fold-diluted culture supernatants of hybridomas secreting anti-spike mAb were incubated with VSV-SARS-CoV-2 for 1 h at 37°C. VeroE6/TMPRSS2 cells were inoculated with the mAb-treated VSV-SARS-CoV-2 and incubated at 37°C under 5% CO_2_. At 24 h post-infection, the infectivity of the VSV-SARS-CoV-2 was determined by measuring the luciferase activity using the Luciferase Assay System (Promega).

### Authentic SARS-CoV-2 neutralization assay

The test mAb was serially diluted with DMEM containing 2% FBS, 100 units/mL penicillin (nacalai tesque), and 100 µg/mL streptomycin (nacalai tesque). Sixty microliters of diluted antibody was mixed with an equal volume of virus (2.4 × 10^3^ PFU/mL). After incubation of the mixture at 37°C for 1 h, 100 µL of the mixture (containing 100 PFU of virus) was inoculated into confluent VeroE6/TMPRSS2 cells in a 6-well plate for 1 h at 37°C under 5% CO_2_. After washing the cells once, the cells were overlaid with 2 mL of DMEM containing 1% Agar (Lonza), 5% FBS, and antibiotics. After incubation at 37°C under 5% CO_2_ for 60 h, the cells were fixed with 10% formalin neutral buffer solution and then stained with 0.1% crystal violet.

### Analysis of viral escape mutants from neutralization antibodies

Serially fivefold diluted mAbs starting at 100 µg/mL were prepared in 500 µL of DMEM containing 2% FBS, 100 units/mL penicillin (nacalai tesque) and 100 µg/mL streptomycin (nacalai tesque). SARS-CoV-2 at 0.1 MOI (4 × 10^4^ PFU) or 2 MOI (8 × 10^5^ PFU) in 500 µl of DMEM containing 2% FBS, 100 units/mL penicillin, and 100 µg/mL streptomycin was mixed with each antibody diluent and incubated at 37°C for 1 h. After the incubation, the mixture was added to VeroE6/TMPRSS2 cells in a 12-well plate and incubated for 96 h at 37°C under 5% CO_2_. The culture supernatants were collected from the wells with the highest antibody concentration that showed an obvious cytopathic effect. For a second round of selection, collected supernatants from cells infected starting at 0.1 MOI or 2 MOI were diluted 1,000- or 250-fold, respectively, in 500 µL of DMEM containing 2% FBS, 100 units/mL penicillin, and 100 µg/mL streptomycin and passaged under the same condition as a first round. Again, the supernatants were collected from the wells with the highest antibody concentration that showed an obvious cytopathic effect. For a third-round selection, supernatants from cells infected at a starting MOI of 0.1 or 2 were diluted 1,000- or 100,000-fold, respectively, mixed with antibodies and then passaged following the same procedure as that used for the second round. After these three rounds of passaging, culture supernatants were collected from the wells with the highest antibody concentration with an obvious cytopathic effect for virus titration and extraction of the viral RNA.

The viral RNA was extracted from the culture supernatant using a QIAamp Viral RNA Kit (QIAGEN). Then, first-strand cDNA was synthesized using a PrimeScript 1st strand cDNA Synthesis Kit (Takara) with extracted RNA and random hexamer primers by following the manufacturer’s protocol. A gene fragment that covers the SARS-CoV-2 S sequence was amplified as previously described (30), and then the amplified product was directly sequenced in both directions with specific primers by using the ABI PRISM 3130 Genetic Analyzer (Applied Biosystems).

### Animal experiments

Eleven-week-old female BALB/c mice (Japan SLC Inc., Japan) were housed in a BSL-3 facility at the Research Institute for Microbial Diseases, Osaka University, for a 1-week adaptation. To determine the mouse median lethal dose (MLD_50_) of rSARS-CoV-2^MA-10^, BALB/c mice were infected with rSARS-CoV-2^MA-10^ sequentially diluted 10^1^–10^5^ TCID_50_. Four mice in each group were observed for survival until 14 dpi: all mice died by 7 dpi in the 10^4^ and 10^5^ TCID_50_ inoculated groups; three of four mice died in the 10^3^ TCID_50_ inoculated group; one of four mice died in the 10^2^ TCID_50_ inoculated group; and all mice survived in the 10^1^ TCID_50_ inoculated group. Together, the MLD_50_ of rSARS-CoV-2^MA-10^ was determined to be 10^2.5^ TCID_50_. To evaluate the protective efficacy of the antibodies, 1 mg/mouse of mAb or isotype control (Wako, Japan) was administered to mice by i.p. injection, and then the mice were intranasally inoculated with five times the 50% mouse lethal dose (5 MLD_50_; equivalent to 1.6 × 10^3^ TCID_50_/50 µL/mouse for this experiment) of SARS-CoV-2 rMA10 virus under isoflurane anesthesia. At 2 dpi, the same dose of mAb was administered to the mice. Mice were monitored daily for survival and body weight changes. At 6 days post-inoculation, the mice were euthanized to obtain lungs and nasal turbinates for virus titration. Viral titers were determined by use of a plaque assay with Vero-E6/TMPRSS2 cells.

The animal experiments were approved by the Institutional Committee of Laboratory Animal Experimentation of the Research Institute for Microbial Diseases, Osaka University (R03-04-0), and all experiments were carried out according to the guidelines of this committee.

### Cryo-EM specimen preparation and data collection

The solution of Spike^B.1^ complexed with the CSW1-1805 Fab was diluted to 0.5 mg/mL with dilution buffer [50 mM HEPES (pH 7.4) and 200 mM NaCl]. For the CSW2-1353 Fab complexes, the solutions of Spike^B.1^ and CSW2-1353 Fab were diluted to 1.0 and 0.57 mg/mL, respectively, with the same dilution buffer, mixed with the same volume to prepare 0.5 mg/mL Spike^B.1^ with a five-time molar excess of CSW2-1353 Fab, and then incubated on ice for 20 min. For the Spike^C480A^ mutants, the protein solutions were diluted to 0.48 mg/mL with the same dilution buffer.

An epoxidized graphene grid (EG-grid) (42) was used to promote the adsorption of protein particles. Three microliters of 0.01 M NaOH and 1% (vol/vol) epichlorohydrin water solution was applied to the ClO_2_-oxidized graphene grids to prepare EG-grids. Then, 3 µL of the protein solutions was applied to the EG-grids and incubated for 5 min at room temperature. The grids were blotted with a force of −3 and a time of 2 s in a Vitrobot Mark IV chamber (Thermo) equilibrated at 4°C and 100% humidity and then immediately plunged into liquid ethane. Excess ethane was removed with filter paper, and the grids were stored in liquid nitrogen.

All cryo-EM image data sets were acquired by using SerialEM (67) and a JEM-Z300FSC (CRYO ARM 300: JEOL, Japan) operated at 300 kV with a K3 direct electron detector (Gatan, Inc., USA) in CDS mode. The Ω-type in-column energy filter was operated with a slit width of 20 eV for zero-loss imaging. The nominal magnification was 60,000×. Defocus varied between –0.5 and –2.0 µm. Each movie was fractionated into 60 frames (0.0505 s each, total exposure: 3.04 s) with a total dose of 60 e^−^/Å^2^.

### Cryo-EM image processing and model building

The images were processed by using RELION 3.1 (68) or 4.0 (69). Movies were motion corrected by using MotionCor2 (70), and the contrast transfer functions (CTFs) were estimated by using CTFFIND 4.1 (71). Micrographs whose CTF max resolutions were beyond 5 Å were selected. 3D template-based auto-picking was performed for all images by using a map of the spike protein trimer (from our previous data set) as a template, and the particles were extracted with 4 × binning. The particle images were subjected to several rounds of 2D and 3D classification. After the high-quality particles were selected, an initial model was generated and used as a reference for the following 3D classification without applying symmetry. The selected particles were re-extracted without binning; 3D auto-refinement without applying symmetry, soft mask generation, or postprocessing were performed. Then, focused 3D classification without alignment was performed to select best-quality particles and to separate different RBD states (only in the CSW2-1353 Fab complexes and Spike^C480A^ data set). CTF refinement, Bayesian polishing, 3D auto-refinement, and postprocessing were conducted with divided optics groups (500 micrographs per group). Another round of CTF refinement, 3D auto-refinement, and postprocessing were then performed. C3 symmetry was imposed in the CSW1-1805 data set. The final map resolutions (FSC = 0.143) were 3.62, 3.66, 3.97, and 3.04 in the CSW1-1805 Fab complexes, CSW2-1353 Fab complexes (1-up RBD), CSW2-1353 Fab complexes (2-up RBD), and Spike^C480A^, respectively.

Homology models of CSW1-1805 and CSW2-1353 Fab complexes were generated by using SWISS-MODEL (72). The atomic model of Spike^B.1^ was built previously (73), and the homology models of the Fab complexes were manually fitted into the density by using UCSF Chimera (74) and Coot (75), and one round of real space refinement was performed in PHENIX (76). Figures were prepared by using ChimeraX (77) and PyMOL (Schrödinger, LLC). The parameters are summarized in Table S3.

## Data Availability

All unique materials generated in this study are made available from the corresponding author with a completed Material Transfer Agreement upon reasonable request. Cryo-EM density maps are available at EMDB with accession codes EMD-33972 (Spike^B.1^ + CSW1-1805 (3-up RBD)), EMD-33973 (Spike^B.1^ + CSW2-1353 (1-up RBD)), EMD-33974 (Spike^B.1^ + CSW2-1353 (2-up RBD)), and EMD-33975 (Spike^C480A^ (2-up RBD)), respectively. Other data supporting this study are available from the corresponding authors upon reasonable request.

## SUPPLEMENTAL MATERIAL

Supplemental material spectrum.03655-23-s0001.pdf Supplemental Figures S1 to S11, supplemental figure legends, and supplemental Tables S1 to S3.

ASM does not own the copyrights to Supplemental Material that may be linked to, or accessed through, an article. The authors have granted ASM a non-exclusive, world-wide license to publish the Supplemental Material files. Please contact the corresponding author directly for reuse.
