# Supplementary material for: Characterization of a neutralizing antibody that recognizes a loop region adjacent to the receptor-binding interface of the SARS-CoV-2 spike receptor-binding domain
Source: Microbiol Spectr. 2024 Feb 28;12(4):e03655-23. doi: 10.1128/spectrum.03655-23 (PMC10986471; doi:10.1128/spectrum.03655-23)
Supplement: Supplemental material — Supplemental Figures S1 to S11, supplemental figure legends, and supplemental Tables S1 to S3. [file spectrum.03655-23-s0001.pdf]

Supplementary Information

- Supplementary Figures S1–11
- Supplementary Figure legends
- Supplementary Tables S1–3

Fig. S1

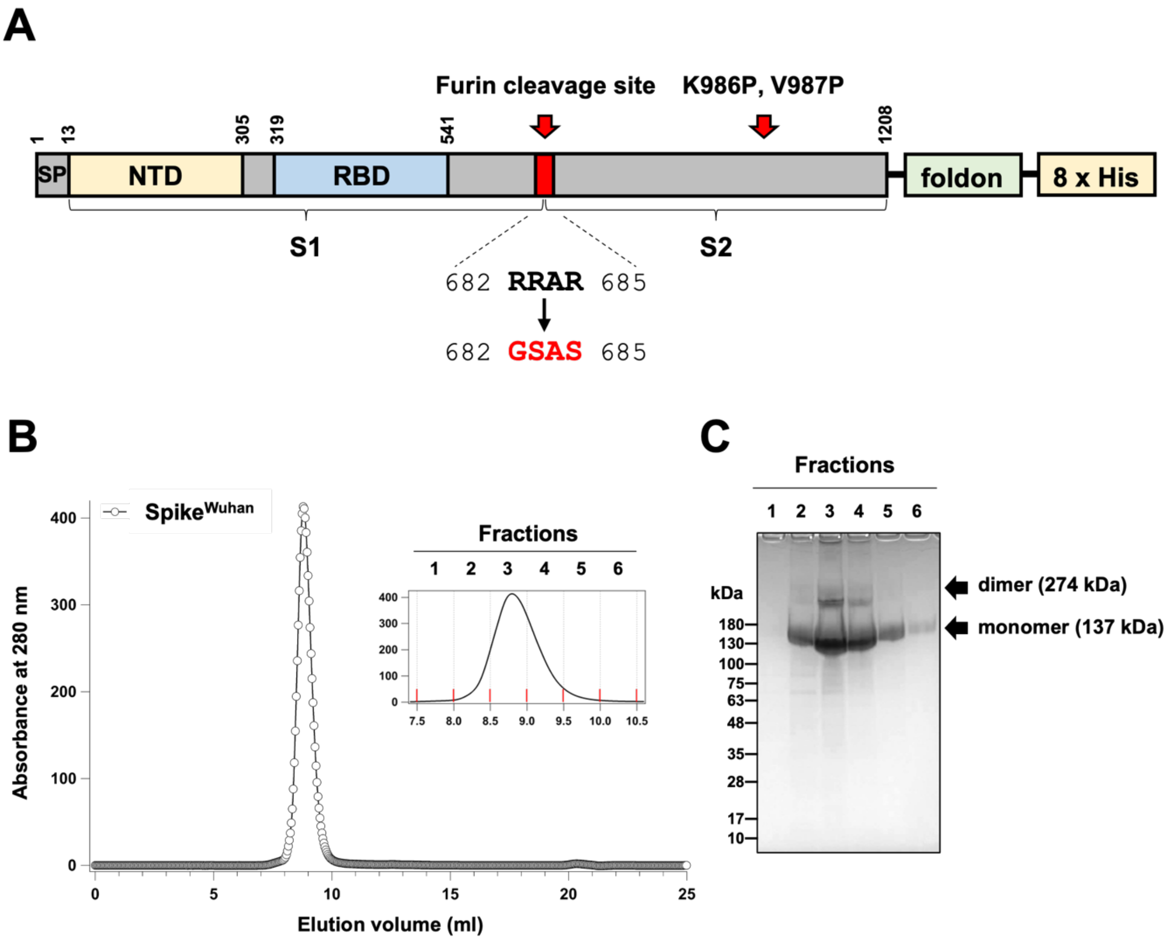
A
Furin cleavage site
K986P, V987P
1
13
305
319
541
1208
SP
NTD
RBD
foldon
8 x His
S1
S2
682 RRAR 685
682 GSAS 685
B
Spike^Wuhan
Absorbance at 280 nm
Elution volume (ml)
Fractions
1 2 3 4 5 6
C
Fractions
1 2 3 4 5 6
kDa
180
130
100
75
63
48
35
28
17
10
dimer (274 kDa)
monomer (137 kDa)

Fig. S2

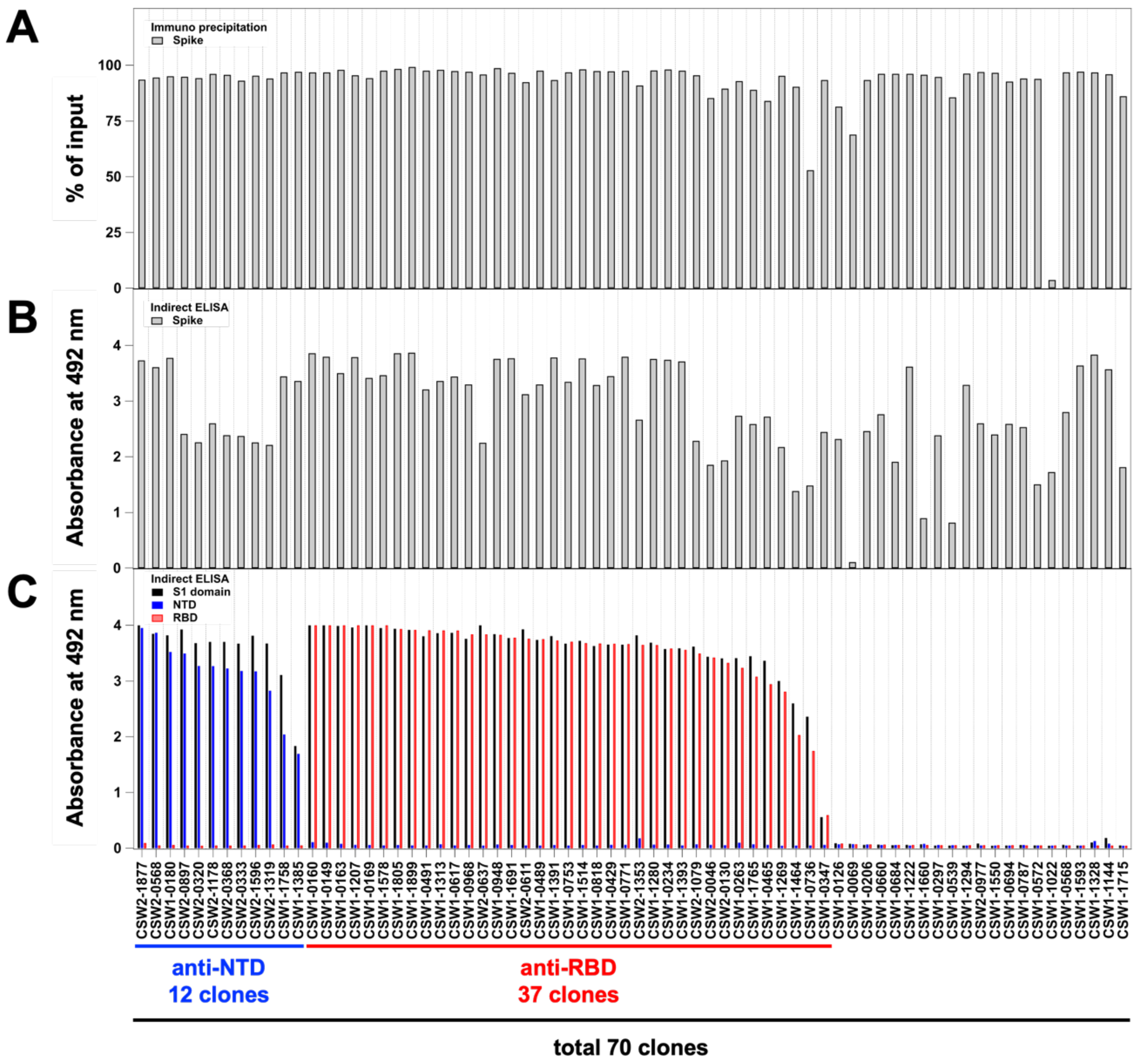

A
Immuno precipitation
Spike
% of input
100
75
50
25
0
B
Indirect ELISA
Spike
Absorbance at 492 nm
4
3
2
1
0
C
Indirect ELISA
S1 domain
NTD
RBD
Absorbance at 492 nm
4
3
2
1
0
CSW2-1877
CSW2-0568
CSW1-0180
CSW2-0897
CSW2-0320
CSW2-1178
CSW2-0368
CSW2-0333
CSW2-1596
CSW2-1319
CSW1-1758
CSW1-1385
CSW1-0160
CSW1-0149
CSW1-0163
CSW1-1207
CSW1-0169
CSW1-1578
CSW1-1805
CSW1-1899
CSW1-0491
CSW1-1313
CSW1-0617
CSW1-0968
CSW2-0637
CSW1-0948
CSW1-1691
CSW2-0611
CSW1-0489
CSW1-1391
CSW1-0753
CSW1-1514
CSW1-0818
CSW1-0429
CSW1-0771
CSW2-1353
CSW1-1280
CSW1-0234
CSW1-1393
CSW2-1079
CSW2-0046
CSW2-0130
CSW1-0263
CSW1-1765
CSW1-0465
CSW1-1269
CSW1-1464
CSW1-0736
CSW1-0347
CSW1-0126
CSW1-0069
CSW1-0206
CSW1-0660
CSW1-0684
CSW1-1222
CSW1-1660
CSW1-0297
CSW1-0539
CSW1-1294
CSW2-0977
CSW1-1550
CSW1-0694
CSW1-0787
CSW1-0572
CSW1-1022
CSW1-0568
CSW1-1593
CSW1-1328
CSW1-1144
CSW1-1715
anti-NTD
12 clones
anti-RBD
37 clones
total 70 clones

Fig. S3

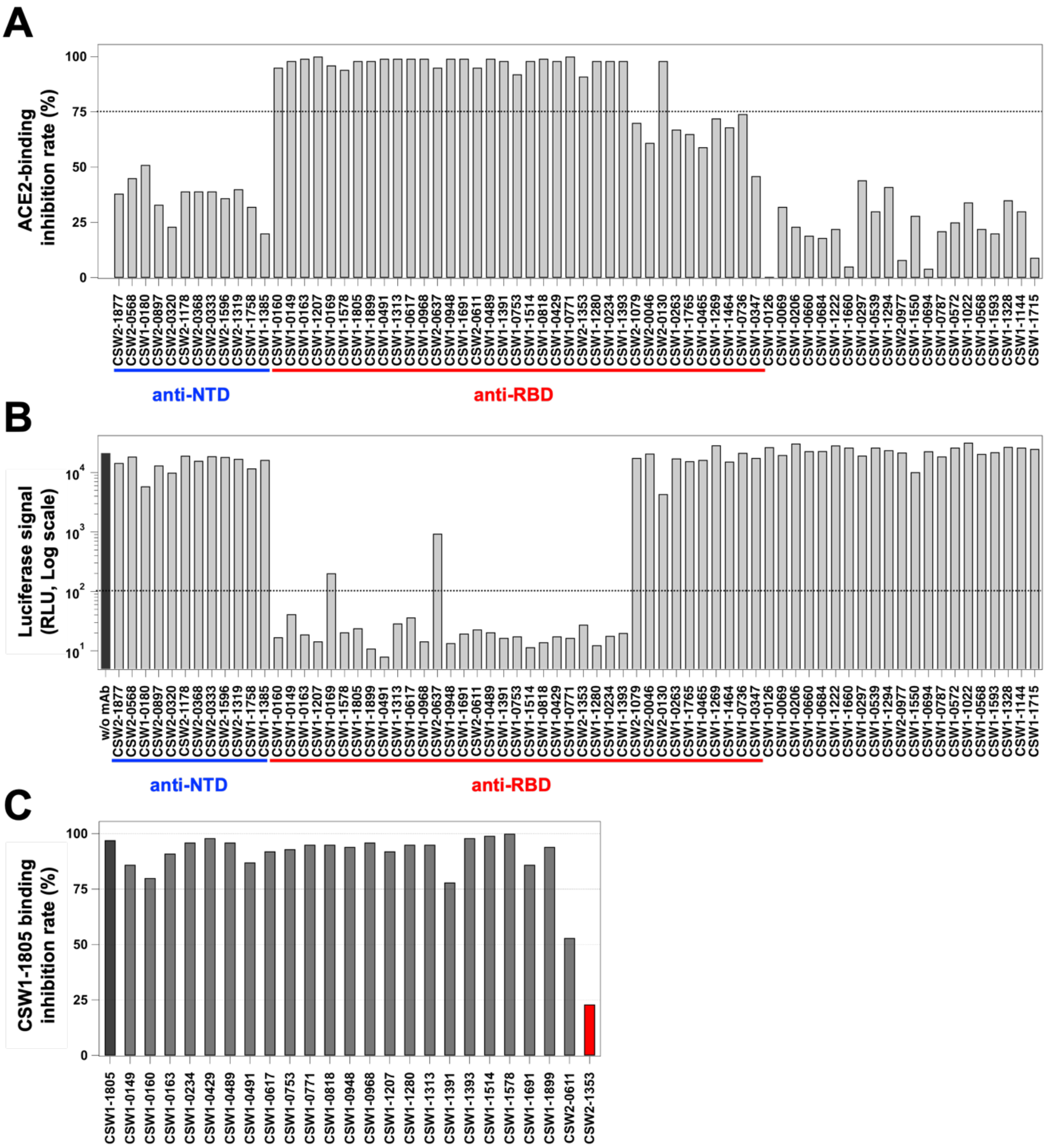
A
ACE2-binding inhibition rate (%)
100
75
50
25
0
anti-NTD
anti-RBD
B
Luciferase signal (RLU, Log scale)
10^4
10^3
10^2
10^1
w/o mAb
anti-NTD
anti-RBD
C
CSW1-1805 binding inhibition rate (%)
100
75
50
25
0
CSW1-1805
CSW1-0149
CSW1-0160
CSW1-0163
CSW1-0234
CSW1-0429
CSW1-0489
CSW1-0491
CSW1-0617
CSW1-0753
CSW1-0771
CSW1-0818
CSW1-0948
CSW1-0968
CSW1-1207
CSW1-1280
CSW1-1313
CSW1-1391
CSW1-1393
CSW1-1514
CSW1-1578
CSW1-1691
CSW1-1899
CSW2-0611
CSW2-1353

Fig. S4

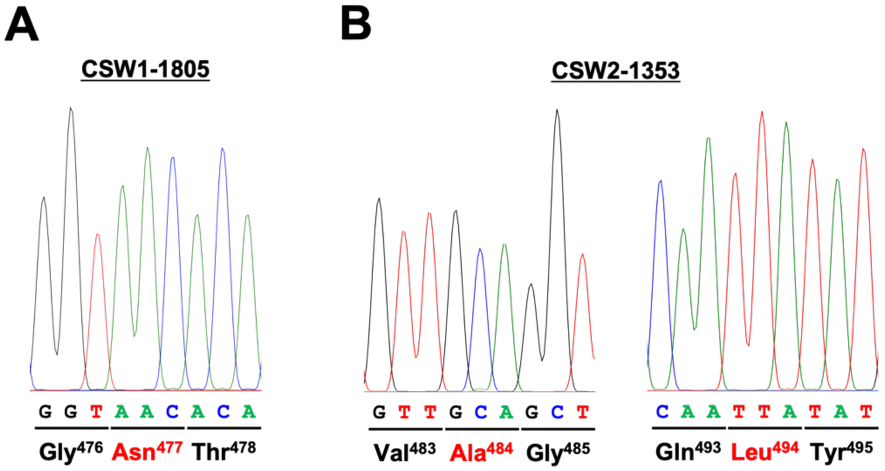
A
B
CSW1-1805
CSW2-1353
G G T A A C A C A
Gly^{476} Asn^{477} Thr^{478}
G T T G C A G C T
Val^{483} Ala^{484} Gly^{485}
C A A T T A T A T
Gln^{493} Leu^{494} Tyr^{495}

Fig. S5

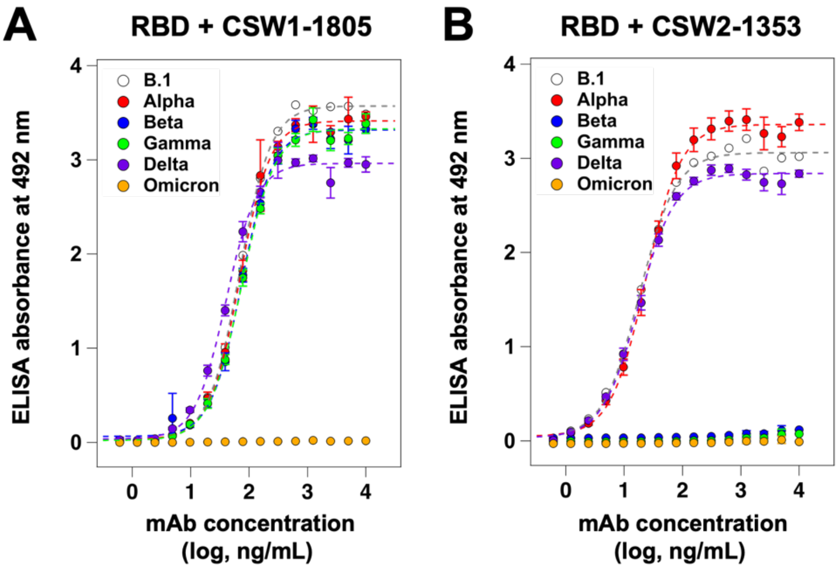

C

```
                        477       484
                         ↓         ↓
    B.1 469 STEIYQAGST PCNGVEGFNC YFPLQSYGFQ PTNGVGYQPY RVVVLSFELL 518
```

```
    B.1 319 RVQPTESIVR FPNITNLCPF GEVFNATRFA SVYAWNRKRI SNCVADYSVL 368
  Alpha 319 ---------- ---------- ---------- ---------- ---------- 368
   Beta 319 ---------- ---------- ---------- ---------- ---------- 368
  Gamma 319 ---------- ---------- ---------- ---------- ---------- 368
  Delta 319 ---------- ---------- ---------- ---------- ---------- 368
Omicron 319 ---------- ---------- D--------- ---------- ---------- 368

    B.1 369 YNSASFSTFK CYGVSPTKLN DLCFTNVYAD SFVIRGDEVR QIAPGQTGKI 418
  Alpha 369 ---------- ---------- ---------- ---------- ---------- 418
   Beta 369 ---------- ---------- ---------- ---------- --------N- 418
  Gamma 369 ---------- ---------- ---------- ---------- --------N- 418
  Delta 369 ---------- ---------- ---------- ---------- ---------- 418
Omicron 369 --L-P-F--- ---------- ---------- ---------- ---------- 418

    B.1 419 ADYNYKLPDD FTGCVIAWNS NNLDSKVGGN YNYLYRLFRK SNLKPFERDI 468
  Alpha 419 ---------- ---------- ---------- ---------- ---------- 468
   Beta 419 ---------- ---------- ---------- ---------- ---------- 468
  Gamma 419 ---------- ---------- ---------- ---------- ---------- 468
  Delta 419 ---------- ---------- ---------- ---R------ ---------- 468
Omicron 419 ---------- ---------- ---------- ---------- ---------- 468
                        477       484
                         ↓         ↓
    B.1 469 STEIYQAGST PCNGVEGFNC YFPLQSYGFQ PTNGVGYQPY RVVVLSFELL 518
  Alpha 469 ---------- ---------- ---------- --Y------- ---------- 518
   Beta 469 ---------- -----K---- ---------- --Y------- ---------- 518
  Gamma 469 ---------- -----K---- ---------- --Y------- ---------- 518
  Delta 469 ---------K ---------- ---------- ---------- ---------- 518
Omicron 469 --------NK -----A---- ----R--S-R --Y------- ---------- 518

    B.1 519 HAPATVCGPK KSTNLVKNKC VNF 541
  Alpha 519 ---------- ---------- --- 541
   Beta 519 ---------- ---------- --- 541
  Gamma 519 ---------- ---------- --- 541
  Delta 519 ---------- ---------- --- 541
Omicron 519 ---------- ---------- --- 541
```

Fig. S6

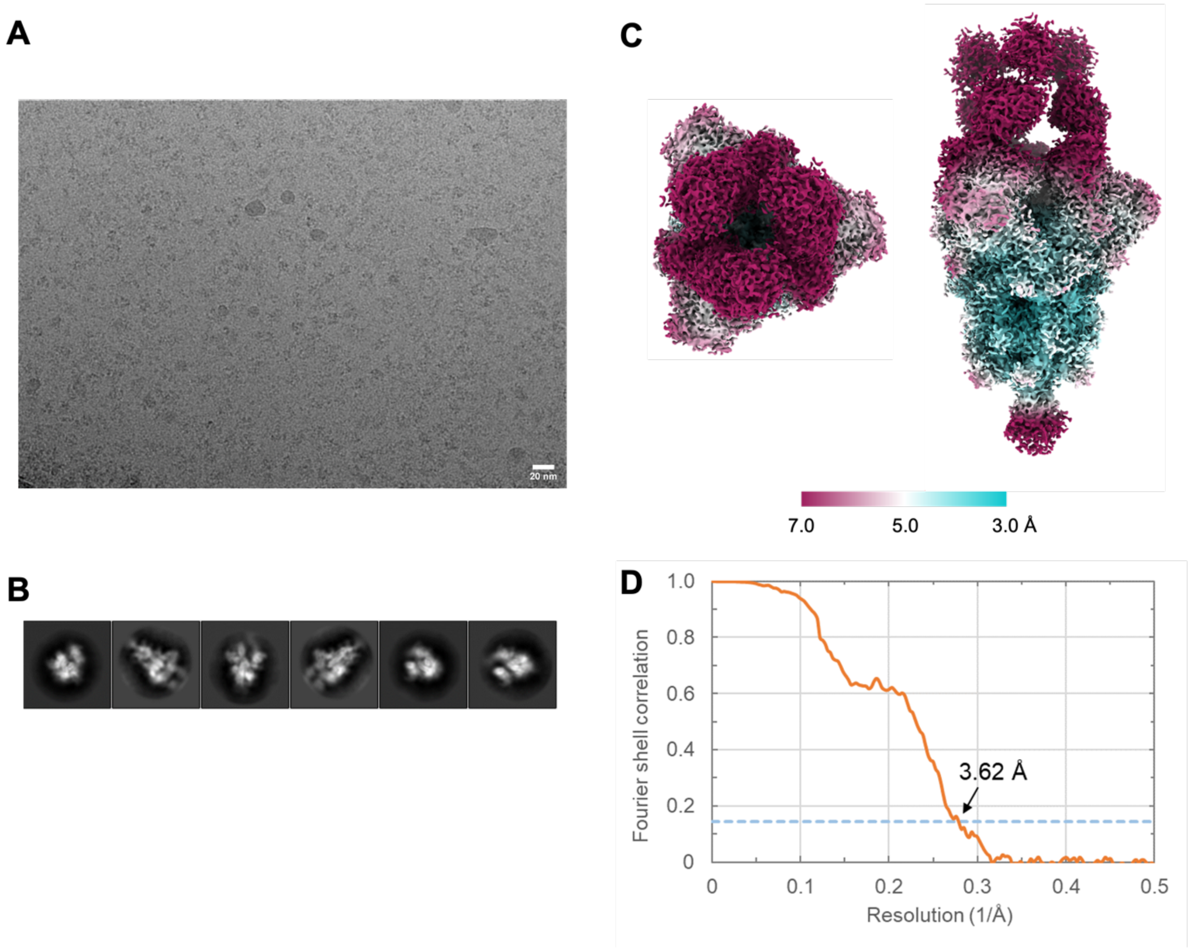
A
20 nm
B
C
7.0
5.0
3.0 Å
D
Fourier shell correlation
1.0
0.8
0.6
0.4
0.2
0
3.62 Å
0
0.1
0.2
0.3
0.4
0.5
Resolution (1/Å)

Fig. S7

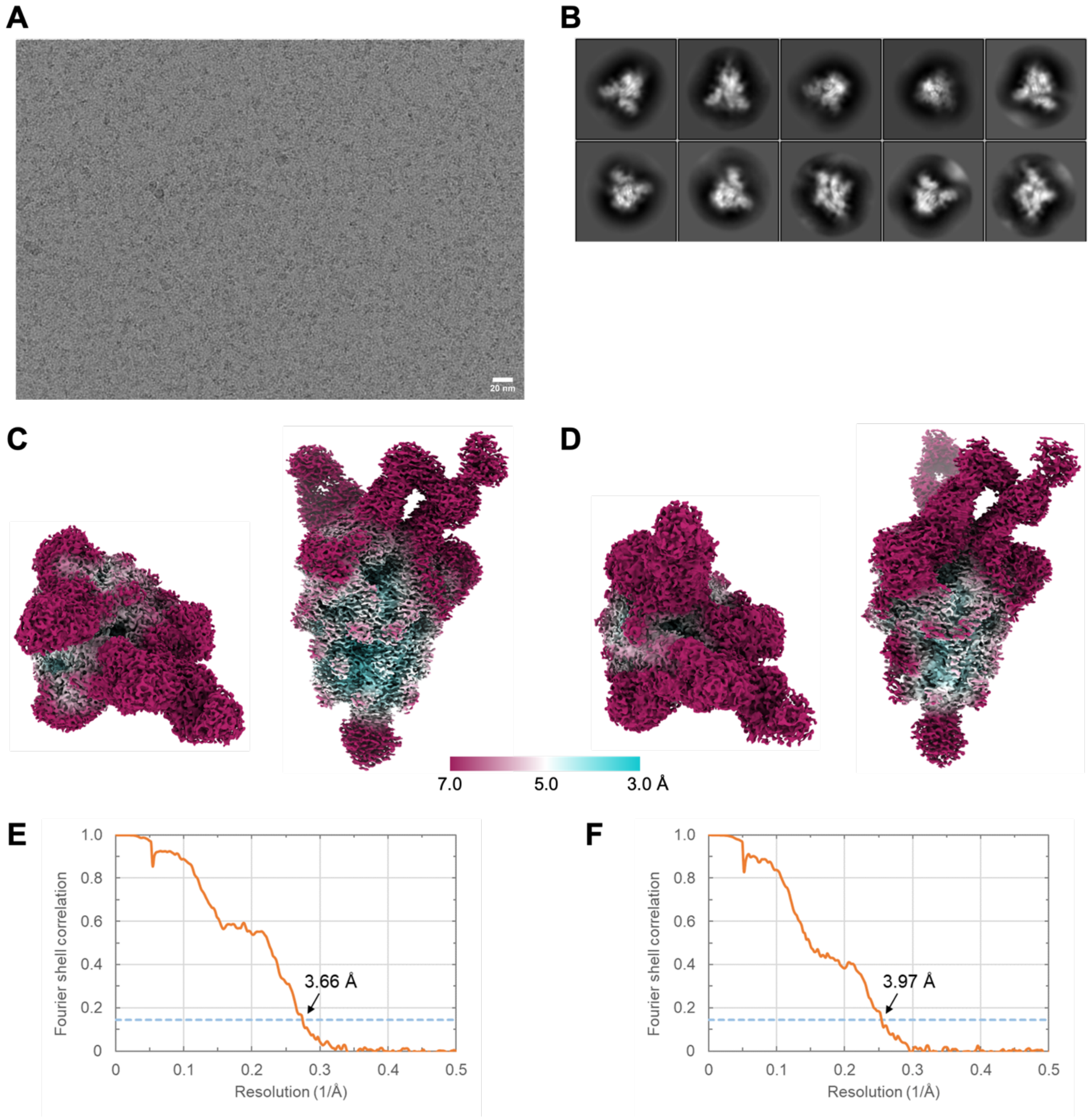
A
B
20 nm
C
D
7.0
5.0
3.0 Å
E
F
Fourier shell correlation
Resolution (1/Å)
1.0
0.8
0.6
0.4
0.2
0
0.1
0.2
0.3
0.4
0.5
3.66 Å
3.97 Å

Fig. S8

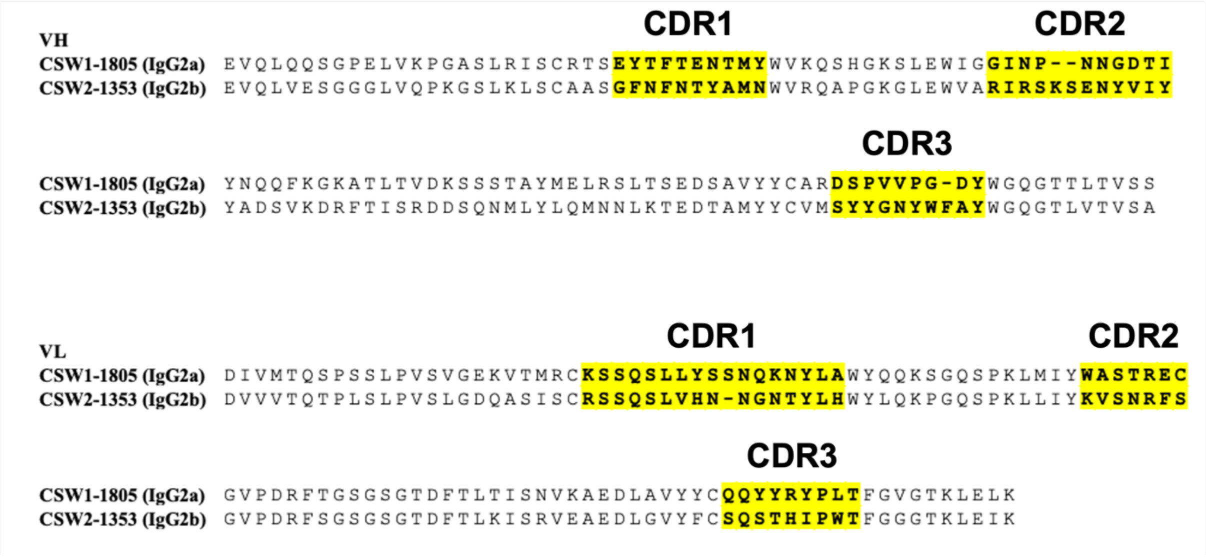
VH
CDR1
CDR2
CSW1-1805 (IgG2a) EVQLQQSGPELVKPGASLRISCRTSEYTFTENTMYWVKQSHGKSLEWIGGINP--NNGDTI
CSW2-1353 (IgG2b) EVQLVESGGGLVQPKGSLKLSCAASGFNFNTYAMNWVRQAPGKGLEWVARIRSKSENYVIY
CDR3
CSW1-1805 (IgG2a) YNQQFKGKATLTVDKSSSTAYMELRSLTSEDSAVYYCARDSPVVPG-DYWGQGTTLTVSS
CSW2-1353 (IgG2b) YADSVKDRFTISRDDSQNMLYLQMNNLKTEDTAMYYCVMSYYGNYWFAYWGQGTLVTVSA
VL
CDR1
CDR2
CSW1-1805 (IgG2a) DIVMTQSPSSLPVSVGEKVTMRCKSSQSLLYSSNQKNYLAWYQQKSGQSPKLMIYWASTREC
CSW2-1353 (IgG2b) DVVVTQTPLSLPVSLGDQASISCRSSQSLVHN-NGNTYLHWYLQKPGQSPKLLIYKVSNRFS
CDR3
CSW1-1805 (IgG2a) GVPDRFTGSGSGTDFTLTISNVKAEDLAVYYCQQYYRYPLTFGVGTKLELK
CSW2-1353 (IgG2b) GVPDRFSGSGSGTDFTLKISRVEAEDLGVYFCSQSTHIPWTFGGGTKLEIK

21 **Fig. S9**

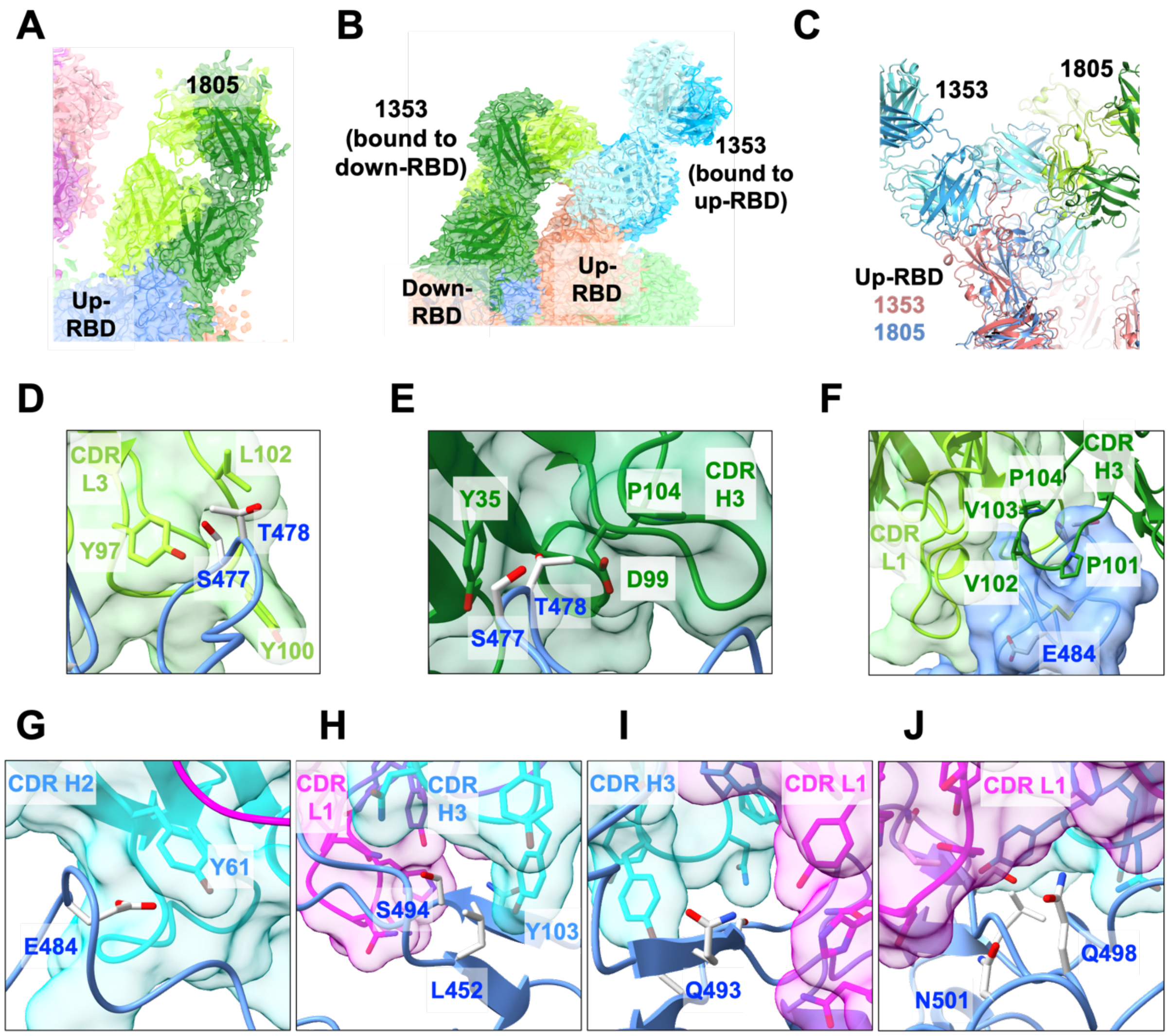

A
1805
Up-RBD
B
1353 (bound to down-RBD)
1353 (bound to up-RBD)
Down-RBD
Up-RBD
C
1353
1805
Up-RBD
1353
1805
D
CDR L3
L102
Y97
T478
S477
Y100
E
Y35
P104
CDR H3
D99
T478
S477
F
CDR H3
P104
V103
CDR L1
V102
P101
E484
G
CDR H2
Y61
E484
H
CDR L1
CDR H3
S494
Y103
L452
I
CDR H3
CDR L1
Q493
J
CDR L1
Q498
N501

22

Fig. S10

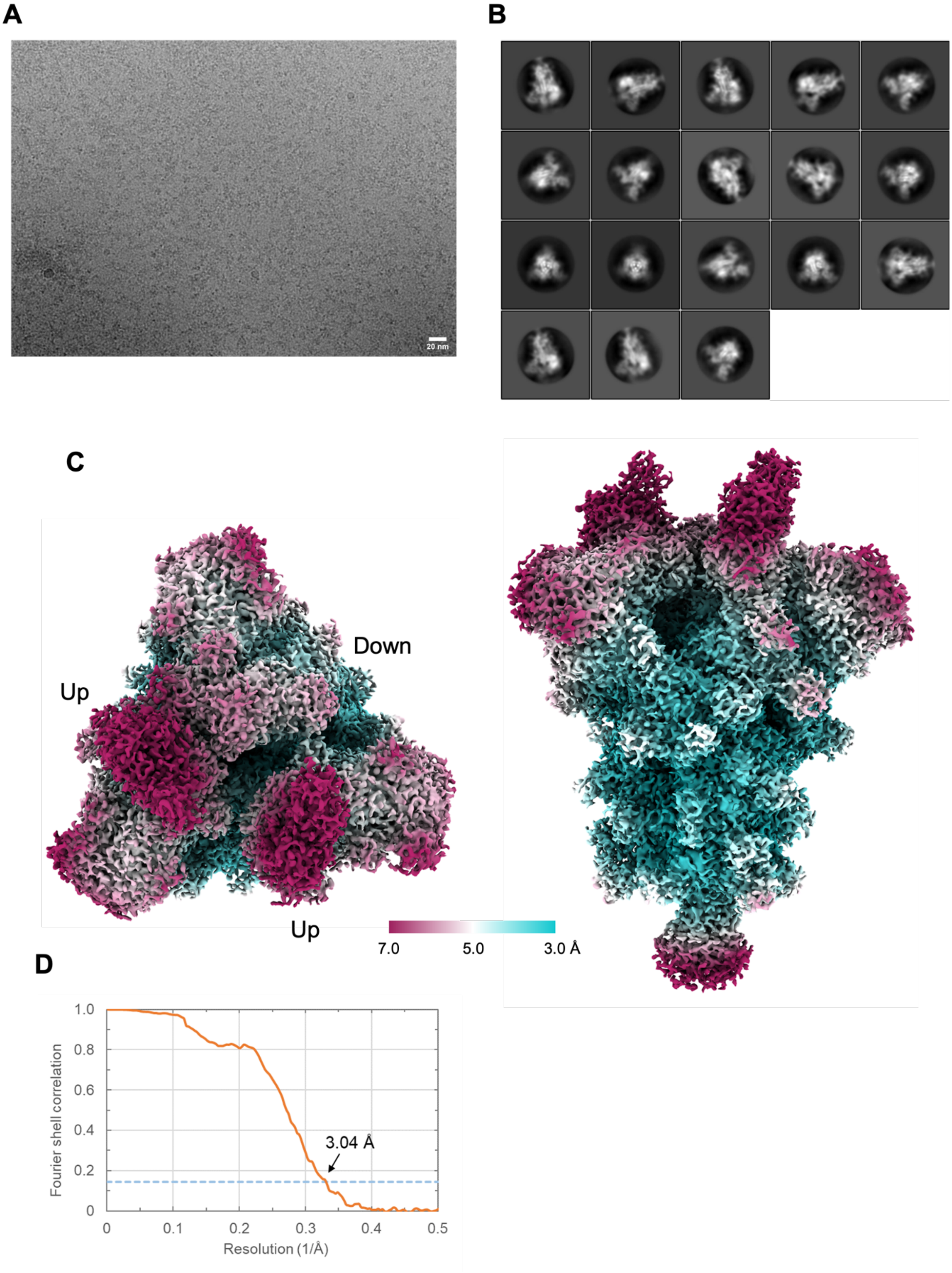
A
20 nm
B
C
Up
Down
Up
7.0
5.0
3.0 Å
D
Fourier shell correlation
1.0
0.8
0.6
0.4
0.2
0
0.1
0.2
0.3
0.4
0.5
3.04 Å
Resolution (1/Å)

Fig. S11

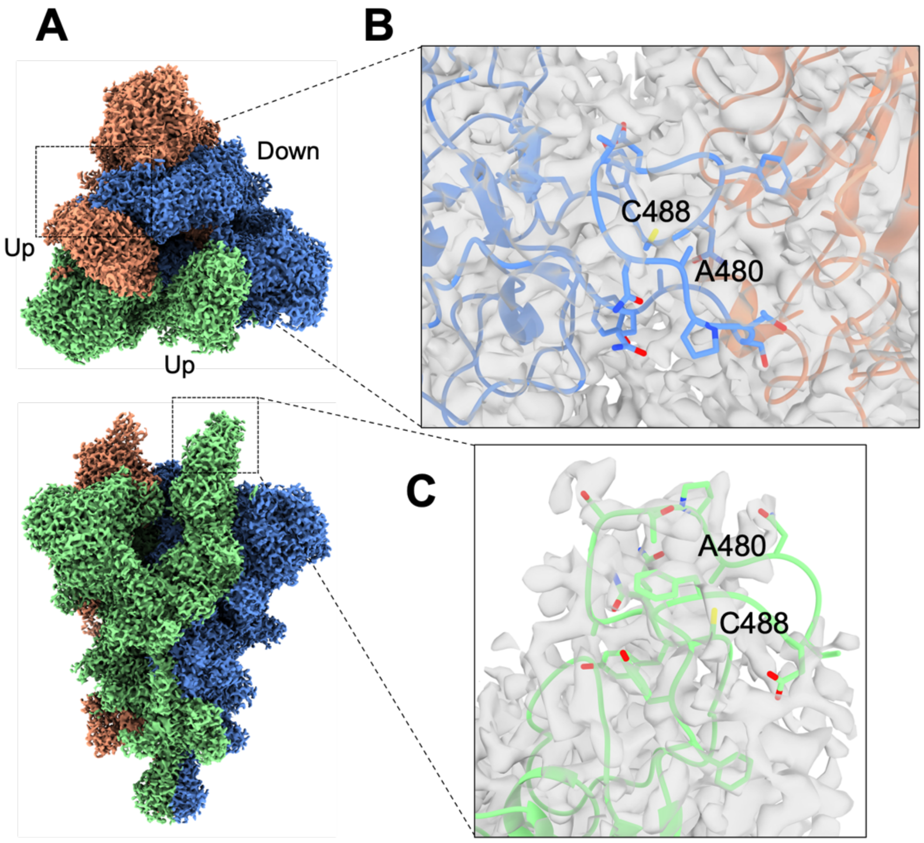

Supplementary Figure Legends

Fig. S1. Preparation of recombinant SARS-CoV-2 Spike protein.

(A) Schematic view of the SARS-CoV-2 spike ectodomain (residues 1–1208) with the GSAS substitution of the furin cleavage site (RRAR; residues 682–685) and the K986P/V987P mutations, followed by the C-terminal, the foldon trimerization motif, and an 8 x His-tag. SP, signal peptide; NTD, N-terminal domain; RBD, receptor-binding domain. **(B)** A representative elution chromatogram of the recombinant spike protein used in this study. The area around the elution peak is shown in the *inset* with the fraction numbers. **(C)** SDS-PAGE of each fraction represented in (B)

Fig. S2. Production of monoclonal antibodies against the SARS-CoV-2 Spike.

(A, B) Screening of hybridoma clones secreting antibody against the SARS-CoV-2 spike. The results obtained by using (A) CELIXSYS^{TM} and (B) indirect ELISA are shown. **(C)** Binding domains of the monoclonal antibodies against the SARS-CoV-2 spike screened by using indirect ELISA. The binding of antibodies against the S1 domain (*black*), NTD, (*blue*), and RBD (*red*) was evaluated.

Fig. S3. Screening of neutralizing antibodies against VSV-pseudotyped SARS-CoV-2.

(A) Inhibition assay for ACE2-Spike binding by the monoclonal antibodies. Biotinylated-ACE2 binding to the spike protein on the ELISA plate treated with or without antibodies was detected. Data are from a single experiment and are presented as the decrease in the percentage of ELISA signal relative to control (without antibody). The dotted line shows a value of 75%. **(B)** Neutralization of VSV-SARS-CoV-2^{Wuhan} by the monoclonal antibodies. VSV-SARS-CoV-2^{Wuhan} was preincubated with or without antibodies. Infectivity of VSV-SARS-CoV-2^{Wuhan} was determined by measuring the luciferase signal. Data are from a single experiment. The dotted line shows a value of 0.5% relative to the luciferase signal without a monoclonal antibody. **(C)** Epitope competition assay. Biotinylated-CSW1-1805 binding to the spike protein on the ELISA plate treated with or without antibodies was detected. Data are from a single experiment and are presented as the decrease in the percentage of ELISA signal relative to control (without antibody).

Fig. S4. Sequence analysis of the SARS-CoV-2 spike gene of escape mutants.

Parts of the sequence electrogram of the SARS-CoV-2 S gene in escape mutants that emerged in the presence of (A) CSW1-1805 or (B) CSW2-1353. The letter of the amino acid at the substituted position is colored in red.

Fig. S5. Binding analysis of CSW1-1805 and CSW2-1353 with the RBD of SARS-CoV-2 variants.

(A, B) Binding curves of (A) CSW1-1805 and (B) CSW2-1353 against RBD$^{B.1}$ were obtained by use of an ELISA. Assays were performed independently three times (means ± SD). The plots were

fitted with a sigmoidal function by using Igor Pro (ver 8.04, Wavemetrics). **(C)** Multiple sequence alignment of the amino acid sequences of the SARS-CoV-2 RBD (a.a. 319–541) of B.1 variants with those of other variants. Only substituted amino acids are shown in the Alpha, Beta, Gamma, Delta, and Omicron variants.

Fig. S6. Cryo-EM analysis of the spike–CSW1-1805 complex.
(A) Typical motion-corrected micrographs (x 600,000). **(B)** Selected 2D class averages aligned in descending order of particle number from left to right and top to bottom. **(C)** Final sharpened maps in two orthogonal views: top view, left panel; and side view, right panel. The local resolution distributions are colored as shown in the color bar. **(D)** FSC curve for the final map. The dashed blue line indicates the FSC = 0.143 criterion.

Fig. S7. Cryo-EM analysis of the spike–CSW2-1353 complex.
(A) Typical motion-corrected micrograph (x 600,000). **(B)** Selected 2D class averages aligned in descending order of particle number from left to right and top to bottom. **(C, D)** Final sharpened maps of (C) 1-up RBD and (D) 2-up RBD datasets in two orthogonal views: top view, left panel; and side view, right panel. The local resolution distributions are colored as shown in the color bar. **(E, F)** FSC curves for the final maps of (E) 1-up RBD and (F) 2-up RBD datasets. The dashed blue lines indicate the FSC = 0.143 criterion.

Fig. S8. Amino acid sequences of variable regions of CSW1-1805 and CSW2-1353.
Amino acid sequences of the heavy (VH) and light (VL) chains of CSW1-1805 and CSW2-1353. Residues in the complementary determinant regions are defined using the AbM definition and highlighted in yellow.

Fig. S9. Close-up views of the spike-Fab complexes.
(A, B) Final sharpened maps of (A) Spike+CSW1-1805 and (B) Spike+CSW2-1353 (1-up RBD) datasets around the Fab molecules. The map regions corresponding to each chain are colored differently (in the same color as Figure 3A, B). Model structures of Fabs were generated by using SWISS-MODEL and fitted into the maps. **(C)** Structural comparison of the up-RBDs bound to CSW2-1353 and CSW1-1805. The coloring is the same as in (A) and (B). **(D–J)** The interface between (D–F) the up-RBD and CSW1-1805 Fab or (G–J) the down-RBD and CSW2-1353 Fab. The RBD is colored in blue, the heavy and light chains of CSW1-1805 in dark green and light green, respectively, and the heavy and light chains of CSW1-1353 in cyan and magenta, respectively.

Fig. S10. Cryo-EM analysis of the spike C480A mutant.
(A) Typical motion-corrected micrograph (x 600,000). **(B)** Selected 2D class averages aligned in descending order of particle number from left to right and top to bottom. **(C)** Final sharpened maps

in two orthogonal views: top view, left panel; and side view, right panel. The local resolution distribution is colored as shown in the color bar. **(D)** FSC curve for the final map. The dashed blue line indicates the FSC = 0.143 criterion.

Fig. S11. Characterization of the spike C480A mutant.

(A) Final sharpened maps in two orthogonal views: top view, top panel; and side view, bottom panel. The map regions corresponding to each protomer in the spike protein are colored in blue, red, and green, respectively. **(B)** Close-up view of the ridge region in down-RBD. Model of the spike trimer extracted from the spike-VHH complex structure (PDB: 7VQ0), modified, and fitted into the maps. **(C)** Close-up view of the ridge region in up-RBD.

Table S1. Affinity data ($K_{d,\ app}$ values) of neutralizing antibodies against the RBD of SARS-CoV-2 variants as determined by use of an ELISA[a]

mAb	Recombinant RBD of:					
	B.1	Alpha	Beta	Gamma	Delta	Omicron
CSW1-1805	67.9 ng/ml (4.53×10^{-10} M)	67.8 ng/ml (4.52×10^{-10} M)	72.2 ng/ml (4.82×10^{-10} M)	74.2 ng/ml (4.94×10^{-10} M)	39.3 ng/ml (2.62×10^{-10} M)	N.D. [b]
CSW2-1353	17.7 ng/ml (1.18×10^{-10} M)	22.8 ng/ml (1.52×10^{-10} M)	N.D.	N.D.	17.2 ng/ml (1.14×10^{-10} M)	N.D.

[a] Sequentially diluted mAb (0–10 μg/ml) was incubated with the RBD of SARS-CoV-2 variants coated on an ELISA plate. RBD-binding mAb was detected by use of HRP-conjugated anti-mouse IgG; obtained signals for each concentration of mAb were plotted. To calculate the $K_{d,\ app}$, the plots were fitted with a sigmoidal function using Igor Pro (ver 8.04, Wavematrics). [b] N.D., no detectable binding under this experimental condition (detection limit is $K_{d,\ app}$ = 10 μg/ml)

Table S2. PRNT50 values of neutralizing antibodies against authentic SARS-CoV-2 isolates as determined in a plaque reduction assay [a]

mAb	Wuhan	Alpha	Beta	Gamma	Delta
CSW1-1805	4.05 ng/ml<br>(27.0 pM)	1.89 ng/ml<br>(12.6 pM)	1.00 ng/ml<br>(6.68 pM)	1.80 ng/ml<br>(12.0 pM)	26.4 ng/ml<br>(176 pM)
CSW2-1353	14.1 ng/ml<br>(93.8 pM)	200 ng/ml<br>(1330 pM)	N.D. [b]	N.D.	10.9 ng/ml<br>(72.7 pM)

[a] An authentic SARS-CoV-2 isolate (100 PFU) was incubated with sequentially diluted mAb (0–50 μg/ml) and then inoculated into VeroE6/TMPRSS2 cells. Plaques in each mAb concentration were counted and the reduction in plaque number against control (without mAb) was plotted. PRNT50 was determined by fitting of the plot with a sigmoidal function using Igor Pro (ver 8.04, Wavematrics). [b]N.D., no detectable binding under this experimental condition (detection limit is PRNT50 = 50 μg/ml)

Table S3. Cryo-EM data collection and image processing

<table>
<tr><th>Dataset</th><th>Spike$^{B.1}$
+ CSW1-1805
(3-up RBD)</th><th>Spike$^{B.1}$
+ CSW2-1353
(1-up RBD)</th><th>Spike$^{B.1}$
+ CSW2-1353
(2-up RBD)</th><th>Spike^{C480A}
(2-up RBD)</th></tr>
<tr><td>EMDB accession no.</td><td>EMDB-33972</td><td>EMDB-33973</td><td>EMDB-33974</td><td>EMDB-33975</td></tr>
<tr><td>Magnification</td><td>60,000</td><td colspan="2">60,000</td><td>60,000</td></tr>
<tr><td>Voltage (kV)</td><td>300</td><td colspan="2">300</td><td>300</td></tr>
<tr><td>Electron exposure ($e^{-}/Å^2$)</td><td>60</td><td colspan="2">60</td><td>60</td></tr>
<tr><td>No. of frames per movie</td><td>60</td><td colspan="2">60</td><td>60</td></tr>
<tr><td>Defocus range (μm)</td><td>−0.5 to −2.0</td><td colspan="2">−0.5 to −2.0</td><td>−0.5 to −2.0</td></tr>
<tr><td>Pixel size (Å)</td><td>0.860</td><td colspan="2">0.884</td><td>0.861</td></tr>
<tr><td>Symmetry imposed</td><td>C3</td><td>C1</td><td>C1</td><td>C1</td></tr>
<tr><td>Micrographs used (no.)</td><td>6,136</td><td colspan="2">3,621</td><td>5,695</td></tr>
<tr><td>Optics group (no.)</td><td>13</td><td colspan="2">8</td><td>11</td></tr>
<tr><td>Initial particle images (no.)</td><td>2,445,672</td><td colspan="2">1,246,763</td><td>1,758,408</td></tr>
<tr><td>Final particle images (no.)</td><td>61,761</td><td>69,304</td><td>44,672</td><td>51,201</td></tr>
<tr><td>Map resolution (Å)</td><td>3.62</td><td>3.66</td><td>3.97</td><td>3.04</td></tr>
<tr><td>FSC threshold</td><td>0.143</td><td>0.143</td><td>0.143</td><td>0.143</td></tr>
<tr><td>Map resolution range (Å)</td><td>3.16–15.1</td><td>3.18–16.9</td><td>3.50–22.0</td><td>2.72–12.2</td></tr>
</table>
